# Multi-objective stochastic scheduling of inpatient and outpatient surgeries

**DOI:** 10.1007/s10696-024-09542-0

**Published:** 2024-06-12

**Authors:** Ambrogio Maria Bernardelli, Lorenzo Bonasera, Davide Duma, Eleonora Vercesi

**Affiliations:** 1 Dipartimento di Matematica “Felice Casorati”, Università degli Studi di Pavia, via Adolfo Ferrata, 5, 27100 Pavia, Italy; 2 Faculty of Informatics, Istituto Dalle Molle di Studi sull’Intelligenza Artificiale (IDSIA USI-SUPSI), Università della Svizzera Italiana, Via La Santa, 1, 6900 Lugano, Switzerland

**Keywords:** Operating room scheduling, Stochastic optimization, Multi-objective optimization, Inpatients, Outpatients

## Abstract

With the advancement of surgery and anesthesiology in recent years, surgical clinical pathways have changed significantly, with an increase in outpatient surgeries. However, the surgical scheduling problem is particularly challenging when inpatients and outpatients share the same operating room blocks, due to their different characteristics in terms of variability and preferences. In this paper, we present a two-phase stochastic optimization approach that takes into account such characteristics, considering multiple objectives and dealing with uncertainty in surgery duration, arrival of emergency patients, and no-shows. Chance Constrained Integer Programming and Stochastic Mixed Integer Programming are used to deal with the advance scheduling and the allocation scheduling, respectively. Since Monte Carlo sampling is inefficient for solving the allocation scheduling problem for large size instances, a genetic algorithm is proposed for sequencing and timing procedures. Finally, a quantitative analysis is performed to analyze the trade-off between schedule robustness and average performance under the selection of different patient mixes, providing general insights for operating room scheduling when dealing with inpatients, outpatient, and emergencies.

## Introduction

With the advancement of surgery and anesthesiology in recent years, surgical clinical pathways have changed significantly, with an increase in outpatient surgeries (Quemby and Stocker 2014). While inpatients are admitted to the hospital by planning an overnight and a certain Length-of-Stay (LoS) in the ward, outpatients require low-complexity surgery and have a state of health that allows them to be discharged within a few hours, except for complications. Although the main difference between inpatient and outpatient care lies in the duration of the stay after the end of the surgery, this two classes of patients have also different characteristics in terms of variability, resources, and needs. For instance, while inpatients have a higher uncertainty in the surgery duration due to the average more complex procedures, outpatients register a higher rate of late cancellations or no-shows. Consequently, in operational contexts dealing with both types of patients by sharing the same Operating Rooms (ORs), these differences should be considered to ensure an adequate outcome and throughput when the surgical schedule is determined.

The surgical scheduling problem is defined as the ensemble of decisions about surgical procedures to be executed in the operating theater, the resource allocation for their execution, and their sequencing within a certain planning horizon (May et al. 2011). This problem lies at the lowest of three decision levels that can be identified in the whole decision process concerning OR planning and scheduling. At the strategic level, a case mix (i.e., a set of specialties) is defined and a pool of ORs is assigned to it. Then, the tactical level concerns the planning of the OR blocks, which is the decision about which ORs have to be opened in the days of the planning horizon, and the definition of a Master Surgery Schedule (MSS) that assigns OR blocks to specialties. Finally, the schedule of the surgeries and their actual execution are managed at the operational level. A recent literature review of the OR Planning & Scheduling (Harris and Claudio 2022) highlighted the high number of aspects to be considered at the three decision levels, showing an increasing complexity of the modeling in terms of considered decision problems in the studies published in the last years.

From an elective patient perspective (Shehadeh and Zuluaga 2022; Testi et al. 2007), a MSS is given as input and the surgical scheduling is solved by addressing two main subproblems, namely the advance scheduling and the allocation scheduling. Under the adoption of the common block-scheduling strategy, which is the most common setting of the real-world operating theaters (Batun et al. 2011), the advance scheduling consists of an *assignment procedure*, in which patients are selected from the waiting list and assigned to the OR blocks of the planning horizon. In dealing with the assignment procedure, several aspects should be taken into consideration, such as the urgency of the patient from a clinical point of view, the time already spent on the waiting list, and the resources required for the surgery execution compared to those available. The most critical resource in both management and expenditure senses is operating time: given a limited OR capacity, surgeries have to be scheduled in accordance with their Estimated Operating Time (EOT), that is a duration estimated by a physician in accordance with the surgery procedure that has to be performed. At the same time, decision-makers should consider that uncertainty factors could lead to a different realization of the surgical procedures with respect to the planned one. Common practices to alleviate the negative effects of uncertainty are to reserve slack times that make the schedule more robust (Saadouli et al. 2015; Van Riet and Demeulemeester 2015; Venkataraman et al. 2018) or to create tailored mixes of patients scheduled in the same OR blocks (Agrawal et al. 2022; Wang et al. 2022).

The allocation scheduling includes a *sequencing procedure* and a *timing procedure*: in the former the order in which the surgeries have to be executed is decided, while the latter determines a start time for each surgery, that is the moment from which the patient will be available to be operated on. The timing procedure includes the management of possible slack time, which can be distributed between subsequent surgeries and/or allocated at the end of the OR block to deal with the negative impact of uncertainty.

Addressing the three procedures, multiple criteria can be defined to determine the optimal global schedule. From a patient-centered point of view, important objectives to be minimized are (i) the scheduling costs related to patient urgency and indirect waiting times (days spent on the waiting list) expressed as a penalty when their surgery is not assigned to any OR block, (ii) the direct waiting time costs as a function of the time elapsed between the planned and the actual start times, and (iii) the cancellation costs consisting of penalties for surgery cancelled for reasons that do not depend on the patients. As claimed by Wang et al. (2021), studies focusing on inpatient and outpatient settings give different importance to these objectives. While the indirect waiting time is more relevant for inpatients, the direct waiting time has a greater interest in outpatients. In addition, a cancellation of an inpatient surgery would have a negative impact on the occupation of resources in the related ward due to the possible growth of the LoS. From an efficiency perspective, two further important objectives to be minimized are (iv) the idle time, in order to avoid wasting resources and the consequent lengthening of the waiting list, and (v) the overtime, which allows the hospital to contain extra costs. Since the OR Planning & Scheduling literature stressed the strong trade-off among all objectives (Aringhieri and Duma 2017; Aringhieri et al. 2022; Cardoen et al. 2009; Samudra et al. 2016; Van Riet and Demeulemeester 2015; Wang et al. 2021; Zhu et al. 2019), a challenging task for the decision maker is to find a good balancing.

Furthermore, we can identify three factors of uncertainty of paramount importance: (a) the deviation between the EOT and the actual surgery duration, called Real Operating Time (ROT), (b) the possible insertion of emergency patients within the planned OR blocks, and (c) the patients’ no-show. To the best of our knowledge, the surgical scheduling problem has never been solved under objectives (i)–(v) and three uncertainty factors (a)–(c) simultaneously.

In this paper, we present a stochastic optimization approach to investigate the potential of chance-constrained and stochastic programming to efficiently exploit the ORs and to guarantee the management of heterogeneous groups of patients, that is patients with different characteristics (e.g., ROT distribution, no-show rate) and needs (scheduling, waiting time, and cancellation costs). Although no-shows are unpredictable, the rationale behind the choice of considering this factor of uncertainty is that such a phenomenon drastically impacts on idle time, and we guess it could be balanced with (or relieved by) other uncertainty components, such as the arrival of emergency patients or surgery duration longer than the expectation. Another contribution of our study is the development of a flexible decision support tool that can be adopted by practitioners from different operative contexts. According to Wang et al. (2021), a deeper investigation should be made about the simultaneous optimization of: the inpatient and outpatient patient flows, characterized by different characteristics (e.g., predictability of the ROT, no-show rates, and costs); the direct and indirect waiting times of outpatients; elective and non-elective patient flow considering patients’ no-shows. All these research questions can be addressed through a flexible optimization approach as the one proposed in this work.

The contribution of this paper is three-fold. Firstly, from a modeling point of view, we formalize the advance scheduling problem through a new Chance-Constrained Integer Programming (CCIP) model and the allocation scheduling problem through a two-stage Stochastic Mixed Integer Programming (SMIP) model under objectives (i)–(v) and uncertainty factors (a)–(c). Secondly, from a methodological point of view, we propose two new alternative heuristics for the allocation scheduling to the standard Monte Carlo sampling: a SMIP-based approach and a genetic algorithm with a custom encoding. Lastly, we provide a computational analysis that shows the effectiveness of both approaches depending on the instance size, and we analyze and discuss obtained results, providing the reader with managerial insights concerning inpatients and outpatients scheduling.

The paper is organized as follows. Section 2 reports the state of art about the surgical scheduling problem that consider at least two of the three mentioned decision procedures. In Sect. 3, we present the problem statement and the two-phase stochastic optimization framework. In Sect. 4, we propose two novel mathematical programming models for the advance and allocation scheduling. In Sect. 5 we present solution approaches based on Monte Carlo sampling and a metaheuristic to solve the two models. After presenting the experimental setup in Sect. 6, the proposed approaches are analyzed in Sect. 7 and used to provide general insights for operational contexts in which inpatients and outpatients are scheduled within the same operating theater. In Sect. 8, we draw conclusions and further research directions.

## Literature review

Under deterministic settings, several approaches can be found in the literature considering assignment, sequencing, and timing procedures simultaneously. Roshanaei et al. (2020) propose a method based on a branch-and-check decomposition to deal with strategical decisions, tactical decisions, and all three operational procedures simultaneously, maximizing the OR utilization and proposing the consideration of stochasticity as a future research direction. Jebali et al. (2006) present a two-step approach to deal with the assignment procedure and the sequencing procedure, also considering pre-operative and post-operative resources, with the purpose of optimizing idle time, overtime, and direct waiting time. Marques et al. (2014) introduce a genetic algorithm to find a near-optimal solution of the assignment procedure and the sequencing procedure, deciding at the same time the assignment of ORs to specialties, in order to maximize OR utilization and number of scheduled patients.

The inclusion of stochasticity in the decision problem increases its complexity. To the best of our knowledge, no work optimizes all three procedures of the surgery scheduling problem in a unified approach. We can identify two main categories of prior articles that take into account two procedures among the assignment procedure, the sequencing procedure, and the timing procedure. The first category concerns studies dealing only with the allocation scheduling, by considering the assignment of patients to OR blocks as an input to optimize both the sequence and the starting times of the surgeries (Çelik et al. 2023; Denton et al. 2007; Lee and Yih 2014; Mancilla and Storer 2012; Xiao and Yoogalingam 2022). The main objective of papers belonging to this category are the minimization of direct waiting times and the minimization of costs related to overtime and the occupation of the medical staff. Several works take into account constraints about the allocation of physical or human resources for activities concerning anesthesia (Çelik et al. 2023) and post-surgery (Lee and Yih 2014). The second category includes works considering both the advance scheduling and the allocation scheduling, focusing only on the sequencing of the surgeries, without optimizing the starting times. Generally, from a computational complexity perspective, this requires a higher effort because the combination of assignment with sequencing considerably widens the search space. For this reason, we assume that the coordination of pre-operative and post-operative activities can be done ex post without limiting the admissible solutions.

In Table 1 we summarize the papers that lie in this last category, as well as our work, to provide a comparison and to highlight the novelties of this paper.

**Table 1:** Summary of prior works that deal with both the advance scheduling and the allocation scheduling under uncertainty. AP, SP, and TP columns indicate if the assignment, sequencing, and timing procedures are addressed, respectively

Work	AP	SP	TP	Other decisions	Uncert.	Objectives	Methodology
Batun et al. (2011)	$\checkmark$	$\checkmark$	$\times$	OR blocks to be opened Physician assignment	ROTs	Overtime Idle time Financial costs	SMIP
Landa et al. (2016)	$\checkmark$	$\checkmark$	$\times$	Overtime allocation	ROTs	OR utilization Cancellations	SMIP CCIP Metaheuristics
Testi et al. (2007)	$\checkmark$	$\checkmark$	$\times$	MSS	ROTs	Overtime OR utilization Throughput Bed utilization	DES ILP Heuristics
Duma and Aringhieri (2015)	$\checkmark$	$\checkmark$	$\times$	Real-time management	ROTs	Overtime OR utilization Throughput Cancellations Ind. waiting time Ddue date violation	DES Metaheuristics Online
Duma and Aringhieri (2019)	$\checkmark$	$\checkmark$	$\times$	Emergency OR policy Real-time management	ROTs Emergency	Overtime OR utilization Cancellations Iind. waiting time Due date violation	DES Metaheuristics Online
Wang et al. (2022)	$\checkmark$	$\checkmark$	$\times$	Surgery partitioning	ROTs Emergency	Overtime Idle time OR utilization Throughput Cancellations Ind. waiting time Due date violation	DES
Agrawal et al. (2022)	$\checkmark ^{*}$	$\checkmark$	$\checkmark$		ROTs	Idle time Ddir. waiting time	SMIP Heuristics
This work	$\checkmark$	$\checkmark$	$\checkmark$		ROTs Emergency No-shows	Overtime Idle time Cancellations Ind. waiting time Dir. waiting time	SMIP CCIP Metaheuristics

A first stream of works addresses the problem with approaches based on stochastic programming. Batun et al. (2011) propose a two-stage SMIP model for the assignment procedure and the sequencing procedure, which are addressed jointly with the number of OR blocks to be used, adopting a pooling strategy for a flexible assignment of the OR blocks. The physician-patient assignment is taken into account, the considered aspect of uncertainty is the surgery duration, and only facility-centered objectives are defined, that is overtime, idle time, and OR financial costs. The authors claim that the L-shaped method fails for realistic instances and they present several structural properties of the SMIP model that lead to computational advantages. Landa et al. (2016) introduce a two-stage CCIP model, where the assignment procedure is solved at the first stage by defining the OR utilization as objective and by fixing a maximum probability of needing overtime in each OR block, then the sequencing procedure and the overtime allocation are performed by the second stage model by minimizing the number of cancellations. Because of the computational complexity of the two stochastic optimization problems, the authors provide a two-phase metaheuristic based on neighborhood search and Monte Carlo simulation.

Another stream of research combine Discrete Event Simulation (DES) with deterministic optimization and/or online optimization approaches. Testi et al. (2007) address the assignment procedure and the sequencing procedure in the last two phases of their three-phase optimization approach, based on an Integer Linear Programming (ILP) model and three greedy heuristics, respectively. After providing a solution for the MSS and the assignment procedure, a DES model is used to analyze the impact of three simple heuristics for the timing procedure, observing indices such as throughput, overtime, idle time, and bed utilization. DES is also proposed by Duma and Aringhieri (2015) for evaluating the impact of a deterministic metaheuristic for the assignment procedure, when it is used jointly with several sequencing policies and online optimization algorithms. Such an approach focuses on the problem of monitoring the actual execution of the surgery, which requires making real-time decisions to deal with the uncertainty of surgery durations and the consequent dynamicity of the operating theater. The authors compare different configurations of their approach with respect to several performance measures, such as the fraction of patients operated within the time limit, throughput, idle time, overtime, and cancellations. The authors provide a more general analysis in Duma and Aringhieri (2019) by proposing online algorithms for the insertion of emergency patients within the operating theater and by evaluating the impact of operating them in dedicated, flexible, or hybrid ORs. Both the advance scheduling and the allocation scheduling are also considered by Wang et al. (2022), which propose a DES model to analyze the impact of two alternative policies when dealing with inpatients having different levels of surgery duration variability, that is the pooling of surgeries with more or less predictable ROTs or the partitioning of the ORs to be assigned to different groups of patients. The arrival of emergency patients to be operated on within a short time limit has been considered, as well as the impact of the proposed policies on multiple criteria. The authors conclude that partitioning patient into two groups reduces the indirect waiting time of elective patients and increases the OR utilization, at the cost of a slight worsening of the cancellation rate and the emergency patients’ waiting time. A recent work by Agrawal et al. (2022) is placed outside this classification, as it combines the sequencing procedure and the timing procedure with the decision of assigning surgeries to OR blocks. Although this decision falls within the assignment procedure, it does not include the selection of patients within the waiting list since it is given as input. The authors formulate the problem with a SMIP model with an objective function that includes penalties for idle time and direct waiting time, and with the surgery duration as a factor of uncertainty. Due to its complexity, the problem is addressed with heuristic approaches for the patient-OR assignment and the sequencing procedure based on a prioritization that depends on the standard deviation of the ROTs, then they use a Monte Carlo simulation for the timing procedure.

In general, most of the prior studies formulate the advance and/or allocation scheduling with stochastic or robust optimization models. While stochastic optimization is more indicated when the probability distribution used to model the uncertainty is known and reliable, robust optimization is suggested when true distributions are not available. In order to deal with conservative solutions provided by robust optimization, distributionally robust optimization models are proposed in Shehadeh (2022), providing robust patient scheduling over an ambiguity set built on little information about the surgical durations, such as mean and variance. We remark that distributionally robust optimization is recommended when dealing with poor historical data or with rare surgical procedures. However, with the rise of healthcare data accessibility in the last decade, it is possible to incorporate surgical time variability into OR scheduling effectively (e.g., see Azar et al. (2022)). Thus, we assume that sufficient information is available to define the surgery duration distribution of the surgical procedures under consideration. Firstly, approximation methods based on Monte Carlo sampling (Loucks 2022) is proposed to solve the CCIP model for the advance scheduling and the SMIP model for the allocation scheduling. When solving the latter with the Monte Carlo sampling, that is the Sample Average Approximation (SAA), computational complexity issues arise when the number of patients scheduled within the same OR block increases. Therefore, we propose two heuristic approaches for solving the allocation scheduling. The first is called *N*-fold SAA and consists of computing the SAA over a partition of the sample into *N* folds. The second is a genetic algorithm, which is a successful methodology in stochastic combinatorial optimization problems arising in various contexts (Bianchi et al. 2008; Gonçalves and Resende 2011), for which we introduce by introducing a custom encoding and a fitness function computed through a Monte Carlo simulation.

## Problem statement

Let us consider a set of specialties *S* and a MSS fixed at the tactical level. This means that we have a set *J* of ORs are assigned to the specialties $s \in S$ over a certain set of days *K*, which is the planning horizon (e.g., $K = \{1,2,3,4,5\}$ for a workweek). Then, the set of all the OR blocks $B \subseteq J \times K$ under consideration can be partitioned in $\left| S\right|$ subsets $B_s$ assigned to the different specialties $s \in S$. OR blocks are indicated with an ordered pair $(j,k) \in B_s$, where *j* is the identifier of an OR assigned to the specialty *s* on the *k*-th day of the planning horizon. From the starting time of an OR block in $B_s$, the block has an ordinary duration $L_{jk}$ to operate on patients. Let *W* be the set of all elective patients to be scheduled, namely the waiting list, then $W = \bigcup _{s_S} W_s$, where $W_s$ is the set of elective patient of the specialty *s*. For every specialty *s*, we have to select a subset of surgeries in $W_s$ and assign them to a specialty’s OR block. OR blocks can use additional time with respect to the planned ending, that is a limited overtime is available with a certain economic cost.

For each patient $i \in W = \cup _{s \in S} W_s$, some information is known and should be taken into account when the OR schedule is defined: the surgical procedure group, the EOT $\mu _i$, the scheduling cost $c_i^{sched}$ (e.g., the ratio of the waiting time over the maximum time before treatment (Duma and Aringhieri 2019; Valente et al. 2009), the waiting time cost $c_i^{wait}$ (per minute), and the cancellation cost $c_i^{canc}$. During the definition of the surgical schedule, the decision maker can take into account several estimators about stochastic aspects concerning the surgery of the patient *i*, such as the mean (i.e., approximately the average duration of their surgical procedure (Pandit and Carey 2006) and the standard deviation $\sigma _i$, and the probability of no-show $r_i$, that is the no-show rate of previous patients of the same specialty or a general group that could be mined from historical data.

For each specialty *s* and for each OR block $(j,k) \in B_s$, the surgical scheduling defines:

*Advance scheduling*: the set $I_{jk} \subseteq W_{s}$ of scheduled patients (assignment procedure);

*Allocation scheduling*: the sequence in which the patients $i \in I_{jk}$ are scheduled within the OR block (sequencing procedure), and the scheduled start time of each patient $i \in I_{jk}$ (timing procedure).

In this study, we deal with the advance scheduling and the allocation scheduling in two phases, that is pertaining to common practice. In fact, the former is set a few days before the planning horizon, while the latter is decided day-by-day since cancellations and postponed surgeries could disrupt the schedule (Aringhieri and Duma 2015; Wang et al. 2021). For instance, this is the case of patients with comorbidities (Tan et al. 2019) or non-elective patients that arrive during the week and need to be operated on within a few days (called add-on or work-in cases (Van Riet and Demeulemeester 2015)).

We first solve the advance scheduling problem by introducing parameters in order to provide different solutions in terms of robustness and patient mixes. Such solutions will depend on patients’ characteristics, such as their surgery duration distribution and different types of costs. Then, we solve the allocation scheduling for each of them, by providing the best overall solution.

Before presenting the stochastic programming models, we make some assumptions regarding hospital policies and patient characteristics.

**Emergency patients**. We assume that the number of daily emergency patients can not be greater than the number of the OR blocks. This allows us to reasonably assume that for every block only one emergency patient surgery can take place, in such a way to ensure a fair unplanned workload balancing. We observe that when this assumption does not correspond to reality, that is when the emergency patient flow increases, dedicated ORs or hybrid policies are recommended by prior studies (Duma and Aringhieri 2019; Van Riet and Demeulemeester 2015). Emergency patients’ arrival times are modeled through independent and identically distributed (i.i.d.) random variables (r.v.s) with uniform distribution on the OR block opening hours, that is a Poisson process. As soon as an emergency patient arrives, every OR can be assigned to them with the same probability regardless of the specialty: we randomly generate such an assignment since it could depend on exogenous factors to decision making. Then, the surgery of the emergency patient will take place as soon as possible: immediately if the assigned room is available, when the current surgery has ended otherwise. Such an insertion rule is often required for non-elective patients classified as *trauma* or *emergency* (Van Riet and Demeulemeester 2015).

**Surgical teams, beds and other resources**. We assume that ORs are the patient flow’s bottleneck in the considered operative context, then we assume that surgical teams, stay beds, post-anesthesia care units, and other resources are always available when needed. We also assume that each OR block has dedicated resources during its execution.

**Real-time policies**. We consider an operative context in which patients are always operated on according to the OR and the sequence determined by the assignment procedure and the sequencing procedure. We assume that the actual surgery start time can not be anticipated and no-shows are known only at the moment of the scheduled time. In addition to the ordinary duration $L_{jk}$ of the OR blocks, a fixed maximum amount of overtime *H* can be performed. Finally, patients are operated on if and only if the estimated surgery completion time does not exceed the maximum overtime available, otherwise the surgery is postponed to the next planning horizon (Duma and Aringhieri 2019; Landa et al. 2016).

## Mathematical models

**Fig. 1:**
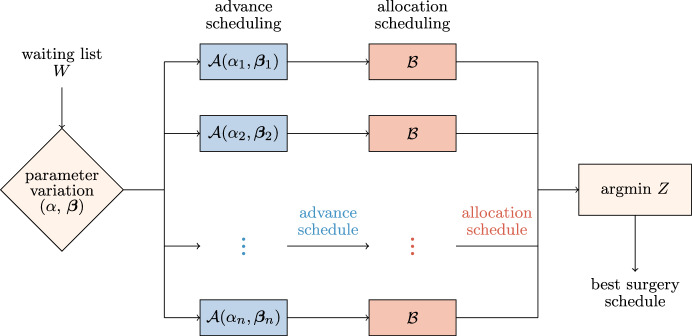
Stochastic optimization framework based on the CCIP model $\mathcal A$ and the SMIP model $\mathcal B$. The robustness parameter $\alpha$ and the patient mix parameter $\varvec{\beta }$ are ranged and the global surgery schedule that minimize the overall objective function *Z* is selected

We propose a two-phase stochastic optimization approach, presenting a stochastic programming formulation for both the advance scheduling and the allocation scheduling, as shown in Fig. 1. Given an instance of the surgical scheduling problem, the proposed framework consists of two stochastic programming models in sequence:

1. A CCIP model $\mathcal {A}(\alpha ,\varvec{\beta })$ provides the solution of the assignment procedure for a certain configuration of model parameters: $\alpha$ defines the level of robustness with respect to the probability of cancellations, while components of parameter vector $\varvec{\beta }$ define the weights of different criteria for patient mixes within the OR blocks;
2. A SMIP model $\mathcal {B}$ uses the previous solution(s) as an input to provide a solution for both the sequencing procedure and the timing procedure.

Although the decomposition of the surgical scheduling into two phases is often performed in literature and real-world, this division could lead to a suboptimal solution of the overall surgical scheduling problem. However, solving the assignment procedure and the sequencing procedure simultaneously is computationally complex (Batun et al. 2011; Landa et al. 2016; Marques et al. 2014), and it would not lead to finding a good quality overall solution within a reasonable running time.

In solving the assignment procedure, one of the most important objectives is the minimization of the patients’ waiting times with respect to their urgency class (Creemers et al. 2012; Testi et al. 2007; Zhu et al. 2019), that is minimizing scheduling costs of non-scheduled patients. Other common objectives in literature are the minimization of idle time and overtime. However, the inherent uncertainty of the operating theater usually leads to significant differences between the planned schedule and the realized one, especially when flexible policies are used to insert emergency patients within the ORs (Duma and Aringhieri 2019). By consequence, while the total scheduling cost can be deterministically computed during the advance scheduling, the other costs (cancellation, waiting, idle time, and overtime) are affected by decisions taken during the allocation scheduling and uncertainty. Therefore, in the proposed framework, we first generate a set of advance schedules in which scheduling costs are minimized by varying the model parameters $\alpha$ and $\varvec{\beta }$. In the second phase, each of them is taken in input by the SMIP model $\mathcal B$ to compute the optimal allocation schedule with respect to the other four costs, which depends on random variables.

We observe that since we assumed that specialties do not share resources, the problem defined by the CCIP model $\mathcal {A}(\alpha ,\varvec{\beta })$ is divided into $\left| S\right|$ independent CCIP models $\mathcal {A}_s(\alpha ,\varvec{\beta })$, that is one for each specialty $s \in S$. Similarly, the problem defined by the SMIP $\mathcal {B}$ can be decomposed into $\left| B\right|$ independent SMIP models $\mathcal {B}_{jk}$, that is one for each OR block $(j,k) \in B$. In the end, the best overall solution is determined, that is the surgery schedule with the minimum sum of the objective functions of the CCIP models $\mathcal {A}_s(\alpha ,\varvec{\beta })$ and the corresponding SMIP models $\mathcal B_{jk}$, is selected.

### Random variables

Before introducing the two models, we define the random vector $\varvec{\xi } = [\varvec{\rho }, \varvec{\delta }, \varvec{\tau }, \varvec{\theta }]$, and the scenario $\omega \in \Omega$, where $\Omega$ is the sample space. Each scenario consists of the realizations of all the following r.v.s., which are independent when not differently specified.

**ROT**. For each patient $i \in I$, their ROT $\rho _i(\omega )$ has a lognormal distribution of mean $\mu _i$ and standard deviation $\sigma _i$.

**Emergency surgery duration**. For each OR block $(j,k) \in B$, the surgery duration $\delta _{jk}$ of the emergency patient assigned to (*j*, *k*) is generated according to a lognormal distribution of mean $\mu ^{em}$ and standard deviation $\sigma ^{em}$ with probability $p^{em} \in [0,1]$, and is equal to 0 with probability $1-p^{em}$ (i.e., no emergency patient is assigned to that OR block).

**Emergency arrival time**. For each OR block $(j,k) \in B$, the emergency arrival time $\tau _{jk}(\omega )$ has uniform distribution in $[0,L_{jk}]$ (if such a surgery exists, otherwise $\tau _{jk}(\omega ) = 0$) and represents the instant from the beginning of the OR block in which the emergency patient arrives. Since we generate such an arrival time independently for each OR, then the overall emergency arrivals within the whole operating theater consist of a Poisson process with rate $\lambda ^{em} = p^{em} \cdot \left| J\right|$ patients per day, that is the interarrival time of emergency patients has an exponential distribution with mean $1/\lambda ^{em}$ over the overall duration of the OR blocks.

**No-show**. For each scheduled patient $i \in I = \cup _{B}I_{jk}$, the r.v. $\theta _i(\omega )$ has Bernoulli distribution of parameter $1 - r_i \in [0,1]$, that is

$$\begin{aligned} \theta _i(\omega ) = {\left\{ \begin{array}{ll} 1 &{} \text {if in scenario } \omega \text { patient } i \text { is available to be operated on,} \\ 0 &{} \text {otherwise (no-show).} \end{array}\right. }. \end{aligned}$$

We remark that all these r.v.s are all realized after the decisions taken at the allocation level, that is no r.v. realizes between advance scheduling and allocation scheduling.

A summary of the notation introduced in the problem statement and in the rest of this section is reported in Table 2.

**Table 2:** Notation (first Latin and then Greek alphabetical order)

*Sets and indices*	
*B*	Set of all OR blocks
$B_s$	Set of all OR blocks assigned by the MSS at the specialty *s*
$i,i',{\hat{i}}$	Elective patients (or their surgery)
*I*	Set of all scheduled patients after the advance scheduling
$I_{jk}$	Set of patients scheduled in the OR block (*j*, *k*) after the advance scheduling
*j*	OR
*J*	Set of all ORs
*k*	Working day
*K*	Set of days of the planning horizon
*s*	Specialty
*S*	Set of all specialties
*W*	Set of all elective patients in the waiting list
$W_s$	Set of elective patients in the waiting list of the specialty *s*
$\omega$	Scenario
$\Omega$	Sample space
*Parameters*	
$b_1,b_2,b_3$	Hierarchy and standardization coefficients
$c^g,c^h$	Idle time cost (per minute) and overtime cost (per minute)
$c_i^{canc}$	Cancellation cost, scheduling cost, and waiting cost of patient *i*
$c_i^{sched}$	Scheduling cost of patient *i*
$c_i^{wait}$	Direct waiting time cost (per minute) of patient *i*
*H*	Maximum overtime per OR block
$L_{jk}$	Ordinary duration of the OR block (*j*, *k*)
$m_s$	Maximum number of patients that can be inserted within the same OR block $B_s$
$M, M_{ii'}$	Big-M
$p^{em}$	Probability of the insertion of an emergency patient in each OR block
$r_i$	Probability of no-show of patient *i*
$\alpha$	Robustness parameter, i.e. approximated probability of cancellation
$\varvec{\beta } = \beta _1,\beta _2,\beta _3$	Proxy weight parameters
$\mu _i$ ($\mu ^{em}$)	Expected surgery duration of elective patient *i* (emergency patient)
$\sigma _i$ ($\sigma ^{em}$)	Standard deviation of surgery duration of elective patient *i* (emergency patient)
*R.v.s*	
$\delta _{jk}$	Actual surgery duration of emergency patient in OR block (*j*, *k*)
$\theta _i$	Patient presence (1 if available in the operating theater, 0 for no-show)
$\varvec{\xi }$	Random vector including all r.v.s
$\rho _i$	Actual surgery duration of elective patient *i*
$\tau _{jk}$	Arrival time of the emergency patient in OR block (*j*, *k*)
*Decision variables*	
$a_i$	Direct waiting time of patient *i*
$c_i ({\hat{c}}_i)$	Completion time of surgery *i* without (by) considering the emergency surgery
*C*	Completion time of the last executed surgery of the OR block
$e_i$	1 if the emergency surgery is subsequent to surgery *i*, 0 otherwise
$g_{jk}$	Idle time in OR block (*j*, *k*)
$h_{jk}$	Overtime in OR block (*j*, *k*)
$o_{ii'}$	1 if surgery $i'$ is subsequent to surgery *i* in the same OR block, 0 otherwise
$q_i ({\hat{q}}_i)$	Start time of surgery *i* without (by) considering the emergency surgery
$t_i$	Scheduled start time of surgery *i*
${\bar{u}}$	Estimated average OR utilization among all OR blocks
$u_{jk}$	Estimated OR utilization of OR blocks (*j*, *k*)
$t_i$	Scheduled start time of surgery *i*
$x_{ijk}$	1 if patient *i* is scheduled in OR block (*j*, *k*), 0 otherwise
$y_i$	1 if the surgery *i* is cancelled due to insufficient residual time, 0 otherwise
$z_i$	Idle time between surgery *i* and emergency surgery if subsequent, 0 otherwise
$\Gamma ^{canc},\Gamma ^{wait},\Gamma ^{time}$	Patient mix proxies

### Advance scheduling: chance constrained Integer programming model

We present the CCIP model for the advance scheduling, that is the assignment procedure. The main modeling aspects of this model are: (i) a chance constraint that sets a minimum level of robustness with respect to cancellations, and (ii) a hierarchical objective function in which three proxies are defined for patient mixes with respect to three different criteria.

The chance constraint is defined by estimating the probability of cancellation under a simplifying assumption, that is the absence of slack time in the actual OR block execution. The value of the model parameter $\alpha \in (0,1)$ indicates that the surgeries assigned to the same OR block must have a probability of exceeding the maximum overtime lower than $\alpha$. In other words, if $\alpha = 0.1$ a probability of at least 90% of not having any cancellations is required.

By varying the parameter $\alpha$, we expect to have an impact on the set *I* of scheduled patients, as a consequence of having more or less possible feasible solutions. Contrariwise, the vector parameter $\varvec{\beta }$ is designed to define a criterion to set a preference in patient mixes, that is the characteristics (surgery duration and costs) of patients assigned to the same OR block.

To this aim, we present a hierarchical objective function where the total scheduling cost is at the upper level and a linear combination of three proxies is at the bottom level. The proxies are defined through the following variables:

$\Gamma ^{canc}$: maximum sum of cancellation costs associated to patient within the same OR block;
$\Gamma ^{wait}$: maximum sum of waiting costs associated to patient within the same OR block;
$\Gamma ^{time}$: sum of differences between the estimated average OR utilization and the estimated OR utilization of all OR blocks (both expressed in minutes).

The first two proxies ($\Gamma ^{canc}$ and $\Gamma ^{wait}$) are introduced to balance the sum of the cancellation and waiting costs among the OR blocks of the same specialty, respectively. The rationale is that advance schedules with all (or many) patients with high cancellation or waiting costs lead to poor allocation schedules. In fact, in this undesired case, some of those surgeries will be inevitably sequenced at the end of the OR blocks with high costs. The third proxy ($\Gamma ^{time}$) is designed to balance the estimated OR utilization between OR blocks, promoting lower idle time and overtime costs.

Let us introduce the decision variables

$$\begin{aligned} x_{ijk} = {\left\{ \begin{array}{ll}1 &{} \text {if the patient } i \in W_s \text { is scheduled into the OR block } (j,k) \in B_s,\\ 0 &{} \text {otherwise}.\end{array}\right. } \end{aligned}$$

The CCIP model $\mathcal {A}_{s}(\alpha ,\varvec{\beta })$ is defined as follows:

1a $$\text{Minimize} \quad \sum\limits_{{i \in W_{s} }} {c_{i}^{{sched}} } \left( {1 - \sum\limits_{{(j,k) \in B_{s} }} {x_{{ijk}} } } \right) + b_{1} \left( {\beta _{1} \Gamma ^{{canc}} + \beta _{2} b_{2} \Gamma ^{{wait}} + \beta _{3} b_{3} \Gamma ^{{time}} } \right)$$

1b $${\text{subject to}}\quad \sum\limits_{{(j,k) \in B_{s} }} {x_{{ijk}} } \le 1,\qquad i \in W_{s},$$

1c $$\sum\limits_{{i \in W_{s} }} {\mu _{i} } x_{{ijk}} \le L_{{jk}} ,\qquad (j,k) \in B_{s},$$

1d $$\begin{aligned} {\mathbb {P}_{\varvec{\xi }}\left[ \delta _{jk} + \sum _{i \in W_s} \theta _i\rho _i x_{ijk} > L_{jk} + H\right] } {\le \alpha , }{\;\; (j,k) \in B_s}{} \end{aligned},$$

1e $$\Gamma ^{{canc}} = \max _{{(j,k) \in B_{s} }} \sum\limits_{{i \in W_{s} }} {c_{i}^{{canc}} } x_{{ijk}},$$

1f $$\Gamma ^{{wait}} = \max _{{(j,k) \in B_{s} }} \sum\limits_{{i \in W_{s} }} {c_{i}^{{wait}} } x_{{ijk}},$$

1g $$\Gamma ^{{time}} = \sum\limits_{{(j,k) \in B_{s} }} | \bar{u} - u_{{jk}} |,$$

1h $$\bar{u} = \frac{1}{{|B_{s} |}}\sum\limits_{{(j,k) \in B_{s} }} {u_{{jk}} } ,$$

1i $$u_{{jk}} = \mathbb{E}_{\varvec{\xi }} \left[ {\delta _{{jk}} } \right] + \sum\limits_{{i \in W_{s} }} {\mathbb{E}_{\varvec{\xi }} } \left[ {\theta _{i} \rho _{i} } \right]x_{{ijk}} ,\qquad (j,k) \in B_{s} ,$$

1j $$x_{{ijk}} \in \{ 0,1\} ,\qquad i \in W_{s} ,\;(j,k) \in B_{s} ,$$

1k $$u_{{jk}} \ge 0,\qquad (j,k) \in B_{s} ,$$

1l $$\Gamma ^{{canc}} ,\Gamma ^{{wait}} ,\Gamma ^{{time}} ,\bar{u} \ge 0.$$

Objective function (1a) is defined as the sum of the total scheduling cost (first summation) and the linear combination of the three proxies, multiplied by the coefficient $b_1$ to create the hierarchy between the two levels. Furthermore, coefficients $b_2$ and $b_3$ are defined to normalize the three proxy variables in order to have the same order of magnitude. To this aim, we first estimate an upper bound of the number of patients that can be scheduled within the same OR block of the specialty $s \in S$

$$m_{s} = \left\lfloor {\frac{{\max _{{(j,k) \in B_{s} }} L_{{jk}} }}{{\min _{{i \in W_{s} }} \mu _{i} }}} \right\rfloor .$$

Then, we define the coefficient

$$b_{2} = \frac{{\max _{{i \in W_{s} }} c_{i}^{{canc}} }}{{\max _{{i \in W_{s} }} c_{i}^{{wait}} }},$$

which balance the first two proxies with respect to their worst case, that is when in an OR block the maximum number of patients is scheduled and they all have the maximum cancellation and waiting cost, respectively. We introduce the coefficient

$$b_{3} = \frac{{m_{s} \max _{{i \in W_{s} }} c_{i}^{{canc}} }}{{\left| {B_{s} } \right|\max _{{(j,k) \in B_{s} }} L_{{jk}} /2}},$$

which is the ratio between the worst case for the first proxy and an approximation of the one for the third proxy (i.e., one half of OR block has full occupation and the other half is empty). The coefficient $b_1$ is finally introduced in such a way to establish a hierarchy in which the sum of the scheduling cost is at the upper level and the proxies are at the bottom level. Let us suppose that the scheduling cost of a surgery $i \in W$ is defined in such a way to have $c_i^{sched} = n \Delta ^{sched}$ for some $n \in \mathbb {N}$. Then, we set

$$b_{1} = \frac{{\Delta ^{{sched}} }}{{1 + m_{s} \max _{{i \in W_{s} }} c_{i}^{{canc}} }},$$

ensuring that proxies cannot lead to choosing as the optimal solution a solution that has a total scheduling cost higher than another feasible solution. Then, by setting the vector of parameters $\varvec{\beta } =(\beta _1,\beta _2,\beta _3)$, with $\beta _1,\beta _2,\beta _3 \in [0,1]$ and $||\varvec{\beta }||_1 = 1$, it is possible to define the weights of the three different proxies in such a way to set the level of preference between the patient mix criteria. We highlight that $\varvec{\beta }$ could or could not have also an impact on the set *I* of scheduled patients. We distinguish two cases: (i) there exists a unique set *I* that minimizes the total scheduling cost in the search space, or (ii) there are two or more sets *I* with the same minimum total scheduling cost. In the former case, the value of $\varvec{\beta }$ does not affect the decision about the surgery to be scheduled, because of the hierarchy of the objective function imposed through $b_1$. In the latter case, different sets *I* could lead to different values of the proxy variables, as a consequence the bottom level of the objective function also acts on the selection of the surgeries. Constraints (1b) impose that each surgery $i \in W_s$ can be scheduled at most once. Constraints (1c) and (1d) are the deterministic and stochastic OR capacity constraints. The former constraints ensure that the sum of the EOT of patients scheduled within an OR block does not exceed the ordinary duration $L_{jk}$. The latter are chance constraints imposing the robustness with respect to the probability of cancellation discussed above. Constraints (1e)–(1f) define the cancellation and waiting time proxies, respectively. Constraints (1g)–(1i) are used to compute the third proxy. In particular, stochastic constraints (1i) estimate the OR utilization $u_{jk}$ of every single OR block $(j,k) \in B_s$ under the same assumption of the chance constraints (1d) and without considering the effect of possible cancellations due to the same considerations about the lack of knowledge of decision that will be taken in the allocation scheduling. Then, constraint (1h) computes the average OR utilization ${\bar{u}}$ over all the estimates and constraint (1g) computes the 1-norm of the vector of differences between the estimated OR utilizations and their mean. Finally, domain constraints are reported in (1j)–(1l).

### Allocation scheduling: two-phase stochastic integer programming model

Given an optimal solution $\varvec{x}^*$ of the CCIP model $\mathcal {A}_s(\alpha ,\varvec{\beta })$, we define the set $I_{jk} = \{i \in W_s \, \mid \, x^*_{ijk} = 1\}$ of all patients scheduled in the OR block $(j,k) \in B_s$. Then, for each OR block we can solve the allocation scheduling problem independently. We propose a two-stage SMIP model $\mathcal {B}_{jk}$ that solves the sequencing procedure and timing procedure simultaneously: at the first-stage (model $\mathcal {B}^I_{jk}$) the expected sum of all the costs is optimized, while in the second-stage (model $\mathcal {B}^{II}_{jk}(\omega )$) we compute the value of several variables in the scenario $\omega \in \Omega$ under the real-time policies defined in Sect. 3. In order to provide a solution for the sequencing procedure and the timing procedure, two types of decision variables are used in $\mathcal {B}^I_{jk}$, that is

$$o_{{ii^{\prime } }} = \left\{ {\begin{array}{*{20}l} 1 \hfill & {{\text{if}}\;{\text{the}}\;{\text{surgery}}\;i^{\prime } \in I_{{jk}} \;{\text{is}}\;{\text{subsequent}}\;{\text{to}}\;{\text{the}}\;{\text{surgery}}\;i \in I_{{jk}} ,} \hfill \\ 0 \hfill & {{\text{otherwise}},} \hfill \\ \end{array} } \right.$$

and the scheduled time $t_i$ of the patient $i \in I_{jk}$ since the OR block start time (min). The first stage of the SMIP model $\mathcal {B}^I_{jk}$ is defined as follows:

2a $$\text{Minimize}\quad {\mathbb {E}_{\varvec{\xi }}[Q(\varvec{o}, \varvec{t};\varvec{\xi }(\omega ))]}$$

2b $${\text{subject to}}\quad {t_i} {\le (L_{jk}-\mu _i)\sum _{i' \in I_{jk} \setminus \{i\}} o_{i'i},}{\;\; i \in I_{jk}},$$

2c $$t_{i} + \mu _{i} \le t_{{i^{\prime } }} + (1 - o_{{ii^{\prime } }} )M_{{ii^{\prime } }} ,\quad i,i^{\prime } \in I_{{jk}} ,i \ne i^{\prime } ,$$

2d $$\sum\limits_{{i \in I_{{jk}} }} {\sum\limits_{{i^{\prime } \in I_{{jk}} \backslash \{ i\} }} {o_{{ii^{\prime } }} } } = \left| {I_{{jk}} } \right| - 1,$$

2e $$o_{{ii^{\prime } }} \in \{ 0,1\} ,\;t_{i} \ge 0,\quad i,i^{\prime } \in I_{{jk}} ,i \ne i^{\prime } .$$

Objective function (2a) is the expected value of the recourse function $Q(\varvec{o}, \varvec{t};\varvec{\xi }(\omega ))$ over the joint distribution of $\varvec{\xi }(\omega )$. Constraints (2b) ensure that each patient *i*’s planned completion time (i.e., the scheduled start time $t_i$ plus the EOT $\mu _i$) does not exceed the OR block capacity $L_{jk}$, forcing the first patient ${\hat{i}}$ to be equal to the OR block start time ($t_{{\hat{i}}} = 0$). Constraints (2c) impose scheduled start times $t_i$ to be consistent with the order defined by $o_{ii'}$ and the planned completion times $t_i + \mu _i$. Constraints (2d) express the logical nature of variables $o_{ii'}$, by guaranteeing to have a complete sequence of all the surgeries in $I_{jk}$. Domain constraints are defined in (2e).

The recourse function $Q(\varvec{o}, \varvec{t};\varvec{\xi }(\omega ))$ corresponds to the objective function of the second-stage stochastic programming model. Thus, the expected value in objective function (2a) is a multidimensional integral of a function that is implicitly defined by a deterministic programming model. The aim of this model is to calculate a weighted sum of costs associated with overtime, idle time, cancellations, and direct waiting time of a scenario $\omega$. This calculation takes into account a set of rules that define all the decisions about overtime allocation, cancellation, and insertion of the (possible) emergency surgery.

**Fig. 2:**
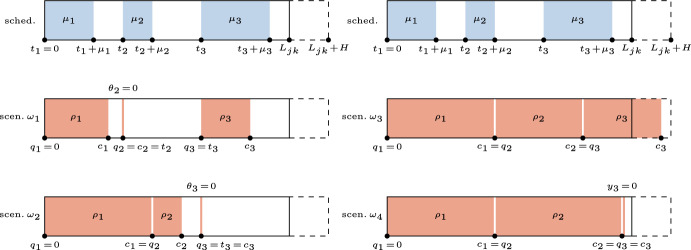
Example of OR block schedule and 4 possible scenarios ($\omega _1,\omega _2,\omega _3,\omega _4 \in \Omega$) such that the emergency surgery is not inserted in the considered OR block ($\delta _{jk} = 0$)

**Fig. 3:**
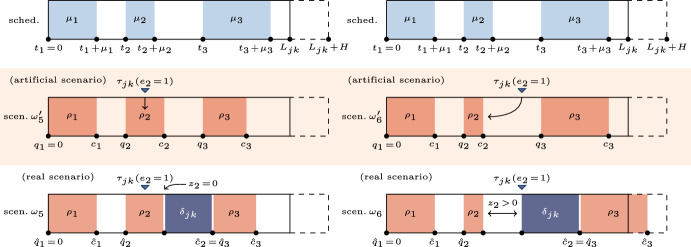
Example of OR block schedule and 2 possible scenarios ($\omega _5,\omega _6 \in \Omega$) such that the emergency surgery is inserted in the considered OR block ($\delta _{jk} > 0$). Artificial scenarios (middle frame) $\omega _5', \omega _6'$ are auxiliary scenarios built with only elective surgeries to identify the surgery *i* to which the emergency surgery is subsequent (indicated with an arrow), and their actual start time, accordingly. When it is not specified, $z_i = e_i = 0$

In Figs. 2 and 3, we report two examples of schedule for a general OR block (*j*, *k*) with 3 elective patients ($i = 1,2,3$) and 6 possible scenarios: 4 different scenarios $\omega _1,\omega _2,\omega _3,\omega _4 \in \Omega$ without the insertion of emergency surgeries ($\delta _{jk} = 0$) are presented in Fig. 2, while 2 further scenarios $\omega _5,\omega _6 \in \Omega$ with the insertion of an emergency surgery ($\delta _{jk} > 0$) are shown in Fig. 3. Since the direct waiting time $a_i$ of the patient *i* is defined as the difference between the actual start time and the scheduled start time $t_i$, we need to compute the first one with respect to all the uncertainty factors that could have an effect on it. For the same reason, we need to determine the actual start and completion times of all patients to compute both the overtime and the idle time, and to establish when surgeries must be canceled.

Firstly, let us consider a general scenario with no emergency surgery to be inserted, such as in Fig. 2. We indicate with $q_i$ the actual start time of the surgery *i*. Such time is equal to $t_i$ if the completion time $c_{i'}$ of the previous surgery does not exceed $t_i$. Otherwise, the surgery of *i* starts as soon as the previous surgery ends, that is at time $c_{i'}$. The completion time is computed as the sum of the actual start time and the ROT ($c_{i'} = q_{i'} + \rho _{i'}$).

For instance, in scenario $\omega _1$ the actual start time of all patients is equal to the scheduled one, since no uncertainty factor compromises the execution of the ex-ante schedule. We remark that, in this scenario, we have a no-show for the patient $i = 2$ (i.e., $\theta _2 = 0$) and, by convention, they will also have a start and completion time set in such a way that they coincide ($q_i = c_i$), although their possible waiting time and cancellation are not considered in terms of cost. An example of direct waiting time greater than 0 can be observed for patient $i = 2$ in scenario $\omega _2$, where the actual duration $\rho _1$ of surgery $i = 1$ deviates significantly from the expected value $\mu _1$, leading to a delay in the completion time $c_1$ and the consequent postponement of the start of the next surgery, which is equal to $c_1$.

Furthermore, we need to introduce the decision variable

$$y_{i} = \left\{ {\begin{array}{*{20}l} 1 \hfill & {{\text{if}}\;{\text{surgery}}\;i\;{\text{is}}\;{\text{executed}}\;{\text{or}}\;{\text{is}}\;{\text{a}}\;{\text{no-show}}} \hfill \\ 0 \hfill & {{\text{otherwise}}} \hfill \\ \end{array} } \right.,\quad i \in I_{{jk}} ,$$

to deal with cases in which the maximum overtime *H* is not sufficient to operate on a patient *i* with respect to their actual start time and the expected surgery duration $\mu _i$. Two opposite situations are presented in scenarios $\omega _3$ and $\omega _4$ depicted in Fig. 2: an amount of overtime equal to $c_3 - L_{jk}$ is used in the former to operate on the patient $i = 3$, while their cancellation ($y_3 = 0$) is necessary in the latter.

To deal with the possible insertion of an emergency surgery (see Fig. 3), we need to introduce two further variables ${\hat{c}}_i$ and ${\hat{q}}_i$ for every patient $i \in I_{jk}$. In these scenarios, $c_i$ and $q_i$ are not the actual completion and start times because the insertion of the emergency surgery could lead to a forward movement of the following elective surgeries. In this case, the actual completion and start time are represented by ${\hat{c}}_i$ and ${\hat{q}}_i$, respectively. By consequence, we use $c_i$ and $q_i$ as auxiliary variables to build an artificial scenario $\omega '$ where the emergency surgery is not inserted, then we define ${\hat{c}}_i$ and ${\hat{q}}_i$ by taking into account the emergency insertion. In scenarios without an emergency patient (i.e., Fig. 2), the actual completion and start times coincide with the corresponding auxiliary variables, that is $c_i = {\hat{c}}_i$ and $q_i = {\hat{q}}_i$.

When the emergency patient arrives (i.e., at time $\tau _{jk}$) they need to be inserted as soon as possible. Two alternative situations could happen.

- A surgery *i* is currently being performed in the OR (e.g., see scenario $\omega _5$ in Fig. 3), then the emergency surgery will start at the completion time $c_i$. By consequence, the idle time $z_i$ between the surgery *i* and the emergency one is equal to 0.
- The OR is currently available (e.g., see scenario $\omega _6$ in Fig. 3), then the surgery can be started immediately. We compute the idle time between the previous elective surgery *i* and the emergency one, which is $z_i = \tau _{jk} - c_i$.

In both situations, we use the auxiliary variable

$$e_{i} = \left\{ {\begin{array}{*{20}l} 1 \hfill & {{\text{if}}\;{\text{the}}\;{\text{emergency}}\;{\text{surgery}}\;{\text{follows}}\;{\text{the}}\;{\text{surgery }}i} \hfill \\ 0 \hfill & {{\text{otherwise}}} \hfill \\ \end{array} } \right.,\quad i \in I_{{jk}} ,$$

to indicate the point in the surgery sequence in which the emergency patient is inserted. Conventionally, we set the variables $z_{i'} = 0$ for the elective surgeries $i' \in I_{jk}$ that do not immediately precede the emergency surgery. Then, we set the variable ${\hat{c}}_i$, associated with the elective surgery *i* such that $e_i = 1$, equal to the completion time of the emergency surgery instead of its own completion time. Finally, we compute the cascade of actual starting times ${\hat{q}}_i$ and completion times ${\hat{c}}_i$ of the following surgeries. We remark that it holds $c_i = {\hat{c}}_i$ and $q_i = {\hat{q}}_i$ for all surgeries that precede the surgery $i'$ such that $e_{i'} = 0$ and for all surgeries in scenarios without the emergency insertion.

The second-stage problem is represented for each scenario by the following programming model $\mathcal {B}_{jk}^{II}(\omega )$:

3a $$\text{Minimize} \quad c^{h} h_{{jk}} + c^{g} g_{{jk}} + \sum\limits_{{i \in I_{{jk}} }} {c_{i}^{{canc}} } (1 - y_{i} ) + \sum\limits_{{i \in I_{{jk}} }} {c_{i}^{{wait}} } a_{i}$$

3b $${\text{subject to}}\quad o_{{ii^{\prime}}} = 1 \Rightarrow q_{{i^{\prime}}} = \max \{ c_{i} ,t_{{i^{\prime}}} \} \wedge \widehat{q}_{{i^{\prime}}} = \max \{ \widehat{c}_{i} ,t_{{i^{\prime}}} \} ,\qquad i,i^{\prime} \in I_{{jk}} ,i \ne i^{\prime },$$

3c $$q_{i} ,\hat{q}_{i} \le M\sum\limits_{{i^{\prime} \in I_{{jk}} \backslash \{ i\} }} {o_{{i^{\prime}i}} } ,\qquad i \in I_{{jk}},$$

3d $$c_{i} = q_{i} + \rho _{i} (\omega )\theta _{i} (\omega )y_{i} + z_{i} + \delta _{{jk}} (\omega )e_{i} ,\qquad i,i^{\prime} \in I_{{jk}} ,i \ne i^{\prime },$$

3e $$\widehat{c}_{i} = \widehat{q}_{i} + \rho _{i} (\omega )\theta _{i} (\omega )y_{i} ,\qquad i,i^{\prime} \in I_{{jk}} ,i \ne i^{\prime },$$

3f $$C \ge \theta _{i} (\omega )(q_{i} + \rho _{i} (\omega )y_{i} ) + z_{i} + \delta _{{jk}} (\omega )e_{i} - (1 - y_{i} )M,\qquad i \in I_{{jk}} ,$$

3g $$C \ge \tau _{{jk}} (\omega ) + \delta _{{jk}} (\omega ),$$

3h $$z_{i} \le Me_{i} ,\;\;i \in I_{{jk}},$$

3i $$\sum\limits_{{i \in I_{{jk}} }} {e_{i} } = 1,\qquad i \in I_{{jk}},$$

3j $$e_{i} = 1 \wedge o_{{ii^{\prime}}} = 1 \Leftrightarrow \widehat{q}_{i} \le \tau _{{jk}} (\omega ) < \widehat{q}_{{i^{\prime}}} ,\quad i,i^{\prime} \in I_{{jk}} ,i \ne i^{\prime },$$

3k $$\left\{ {\begin{array}{*{20}c} {e_{i} = 1} \\ {\tau _{{jk}} (\omega ) > q_{i} + \rho _{i} (\omega )\theta _{i} (\omega )y_{i} } \\ \end{array} } \right. \Rightarrow \begin{array}{*{20}c} {z_{i} = \tau _{{jk}} (\omega ) - } \\ {(q_{i} + \rho _{i} (\omega )\theta _{i} (\omega )y_{i} ),} \\ \end{array} \qquad i \in I_{{jk}},$$

3l $$\theta _{i} (\omega )(q_{i} + \mu _{i} ) \le L_{{jk}} + H \Leftrightarrow y_{i} = 1,\qquad i \in I_{{jk}},$$

3m $$y_{i} \ge 1 - \theta _{i} (\omega ),\qquad i \in I_{{jk}},$$

3n $$a_{i} \ge q_{i} - t_{i} - M(1 - y_{i} \theta _{i} (\omega )),\qquad i \in I_{{jk}},$$

3o $$h_{{jk}} \ge C - L_{{jk}},$$

3p $$g_{{jk}} \ge \max \{ L_{{jk}} ,C\} - \sum\limits_{{i \in I_{{jk}} }} {\rho _{i} } (\omega )\theta _{i} (\omega )y_{i} - \delta _{{jk}} (\omega ),$$

3q $$h_{{jk}} ,g_{{jk}} ,q_{i} ,\hat{q}_{i} ,c_{i} ,\hat{c}_{i} ,C,z_{i} ,a_{i} \ge 0,\;y_{i} ,e_{i} \in \{ 0,1\} ,\qquad i \in I_{{jk}} .$$

Objective function (3a) is multi-objective and includes four terms. The first term $c^h h_{jk}$ is the total overtime cost in the considered OR block (*j*, *k*); the second term $c^g g_{jk}$ is the total idle time cost in the OR block (*j*, *k*); then we consider the sum of cancellation costs $c_i^{canc}$ for canceled surgeries *i* due to insufficient residual time; finally, we consider the sum of waiting costs, computed as the cost per minute $c_i^{wait}$ multiplied by the minutes of direct waiting time $a_i$ of the patient *i*.

Constraints (3b)–(3c) define the actual start times ${\hat{q}}_i$ in the considered scenario and the start time $q_i$ in the corresponding artificial scenario. Constraints (3b) define the actual start times as the maximum between the scheduled start times and the completion times of the previous patients (if they exist). Constraints (3c) impose that the start time of the first surgery is equal to the OR block’s start time, that is 0, while it becomes redundant with respect to (3b) for all other surgeries due to the big-M.

Similarly, variables ${\hat{c}}_i$ and $c_i$ are defined by constraints (3d)–(3e) as the actual completion times in the real scenario and in the artificial scenario, respectively. In both the equalities, the ROT of *i* is summed to the actual start times only if the patient is available to be operated on ($\theta _i = 1$) and is not necessary to cancel their surgery ($y_i = 1$). In the particular case in which the emergency surgery is inserted immediately after the patient *i* ($e_i = 1$), ${\hat{c}}_i$ is defined as the completion time of the emergency patient, to which two further durations are added: the idle time $z_i$ between the elective patient *i* and the emergency patient, and the surgery duration $\delta _{jk}(\omega )$ of the emergency patient inserted in the OR block (*j*, *k*). For instance, in both scenarios $\omega _5$ and $\omega _6$ of Fig. 3 the emergency surgery is subsequent to the elective surgery $i = 2$, then ${\hat{c}}_2 = {\hat{q}}_2 + z_2 + \delta _{jk}$.

Constraints (3f)–(3g) compute the maximum completion time *C*, that is the actual end time of the OR block, remarking that one among constraints (3f) and constraint (3g) is more strict with respect to all the others. There are two possible cases that cause the redundancy of a constraint with respect to the strictest one: (i) the considered surgery is not the last executed in the OR block, and (ii) the considered surgery is not executed. For instance, in scenario $\omega _2$ in Fig. 2 we have $C = {\hat{c}}_2 = c_2$ because when considering the constraint (3f) for $i = 1$, we have that $C \ge {\hat{c}}_1$, but for $i = 2$ it holds that $C \ge {\hat{c}}_2 = c_2 > {\hat{c}}_1 = c_1$. At the same time, we do not consider the completion time of the surgery $i = 3$ due to its no-show and the absence of an emergency patient after it, which leads to the trivial constraint $C \ge 0$ when considering the constraint (3f) for $i = 3$. Similarly, in scenario $\omega _4$ it holds that $C = {\hat{c}}_2 = c_2$ since the cancellation of the patients $i = 3$ enables the big-M when considering the same constraint. Furthermore, constraint (3g) is necessary for the particular case in which the emergency arrival occurs after the auxiliary actual starting time ${\hat{q}}_i$ of the last surgery of the OR block, but it is a no-show or is canceled.

Furthermore, when the emergency patient does not succeed the patient *i* ($e_i = 0$), variable $z_i$ is fixed to 0 by constraints (3h) because there is no idle time to compute between such a surgery and the emergency one (e.g., this is the case of scenario $\omega _5$ in Fig. 3).

Constraints (3i) ensure that the emergency surgery is inserted exactly one time. Constraints (3j) implement the emergency surgery’s insertion policy, that is (i) at the exact moment $\tau _{jk}(\omega )$ of the emergency patient arrival if the OR is available (such as in scenario $\omega _6$ in Fig. 3), (ii) immediately after the surgery currently in progress otherwise (such as in scenario $\omega _5$ in Fig. 3). Constraints (3k) compute the idle time between the emergency surgery and the previous elective surgery *i*, that is equal to 0 when the emergency patient arrives while elective patient *i* is within the OR and they is operated immediately after the end of their surgery.

Constraints (3l) apply the cancellation policy, that is a certain surgery is canceled if and only if its expected completion time (i.e., ${\hat{q}}_i + \mu _i$) would exceed the maximum overtime (i.e., $L_{jk}+H$), then constraints (3m) avoid the cancellation if the patient is a no-show.

Constraints (3n) compute the waiting time of the patient *i* as the difference between the actual and the scheduled start time, using a big-M to set to 0 the waiting time of no-shows and patient whose surgery has been canceled. Constraint (3o) defines the overtime as the difference between the maximum completion time *C* and the OR block ordinary duration $L_{jk}$. Constraint (3p) defines the idle time as the difference between the maximum completion time *C* and the sum of the ROTs of the patients operated on.

All variables are bounded due to domain constraints (3q). All non-trivial constraints presented here in a non-linear form for the sake of summary are reported in the corresponding linear form in Appendix A.

### Overall objective function

We can define the overall objective function of the surgical scheduling problem defined in the stochastic optimization framework at the beginning of this section (see Fig. 1) as follows:

4 $$\begin{aligned} Z = & \sum\limits_{{s \in S}} {\sum\limits_{{i \in W_{s} }} {c_{i}^{{sched}} } } \left( {1 - \sum\limits_{{(j,k) \in B_{s} }} {x_{{ijk}} } } \right) \\ & + \sum\limits_{{(j,k) \in B}} {\mathbb{E}_{\varvec{\xi } }} \left[ {c^{h} h_{{jk}} + c^{g} g_{{jk}} + \sum\limits_{{i \in I_{{jk}} }} {\left( {c_{i}^{{canc}} (1 - y_{i} ) + c_{i}^{{wait}} a_{i} } \right)} } \right], \\ \end{aligned}$$

where variables at the bottom level of the hierarchical objective function of $\mathcal {A}_s(\alpha ,\varvec{\beta })$ are not included since they are only used as proxies to link the two phases of the stochastic optimization framework.

## Methodology

Because of the complex structure of the stochastic process under which the surgical scheduling is executed, closed-form expressions of the chance constraints (1d) of $\mathcal {A}_s(\alpha ,\varvec{\beta })$ and of the stochastic objective function (2a) of $\mathcal {B}_{jk}$ are complex to be defined in an exact way or by introducing a good approximation. For this reason, we propose a Monte Carlo sampling to deal with the chance constraints (1d) for the advance scheduling in Sect. 5.1 and two different versions of the SAA method to deal with the stochastic objective function of the allocation scheduling model in Sect. 5.2. Nevertheless, as we will show in Sect. 6, the combination of the stochastic and computational complexities of $\mathcal {B}_{jk}$ makes the SAA methodology not effective for some instances, because it consists of the solution of a mixed integer linear programming model with a high number of variables. Therefore, in Sect. 5.3 we propose a genetic algorithm with a custom encoding and a fitness function evaluation based on Monte Carlo sampling to find a near-optimal solution with a reasonable computational cost.

### Advance scheduling: Monte Carlo sampling

The CCIP model $\mathcal {A}_s(\alpha ,\varvec{\beta })$ is solved by approximating the chance constraints (1d) and the stochastic constraints (1i) through a Monte Carlo sampling. Firstly, a random sample $\mathcal {S}$ is generated. Then, the probability in (1d) is approximated by

5 $$P_{{jk}} = \frac{1}{{|{\mathcal{S}}|}}\sum\limits_{{\omega \in {\mathcal{S}}}} {1_{{(L_{{jk}} + H, + \infty )}} } \left( {\delta _{{jk}} (\omega ) + \sum\limits_{{i \in W_{s} }} {\theta _{i} } (\omega )\rho _{i} (\omega )x_{{ijk}} } \right),\quad (j,k) \in B_{s} ,$$

where the indicator function $\textbf{1}_{(L_{jk}+ H,+\infty )}$ is used to compute the number of scenarios in which the internal inequality is not satisfied. Similarly, auxiliary variables $u_{jk}$ defined in constraints (1i) are approximated with their sample average

6 $$\tilde{u}_{{jk}} = \frac{1}{{|{\mathcal{S}}|}}\sum\limits_{{\omega \in {\mathcal{S}}}} {\left( {\delta _{{jk}} (\omega ) + \sum\limits_{{i \in W_{s} }} {\theta _{i} } (\omega )\rho _{i} (\omega )x_{{ijk}} } \right)} ,\quad (j,k) \in B_{s} .$$

### Allocation scheduling: SAA and *N*-fold SAA

We propose two alternative SAA methods, that is objective function (2a) computed through a randomly generated sample $\mathcal S$ of size $|\mathcal {S}|$, to find a near-optimal solution of $\mathcal B_{jk}$.

*SAA*:  The SMIP model is solved by computing the classic SAA method (Kleywegt et al. 2002) on $\mathcal {S}$. 
*N*-fold SSA ($SAA_{N}$): The sample $\mathcal {S}$ is partitioned into *N* folds of size $|{\mathcal {S}}| /N$. Then, the stochastic programming is solved by computing the SAA method on each subset $\mathcal {F}_n \subset \mathcal {S},\, n = 1,\ldots ,N$. The solution obtained for each fold $\mathcal F_n$ is then reevaluated on the entire sample $\mathcal {S}$ through an algorithmic straightforward implementation of $\mathcal B_{jk}^{II}$. Finally, the one with the minimum objective function value is selected.

### Allocation scheduling: a genetic algorithm

We present a custom version of the **Biased Random-Key Genetic Algorithm (BRKGA)**, which is an effective method for tackling sequencing problems (Gonçalves and Resende 2011) and we adapt it in order to solve also the timing procedure. In our customization, each solution of the optimization problem consisting of the sequencing procedure and the timing procedure for an OR block (*j*, *k*) is encoded through a vector

$$\varvec{\Gamma } = (\gamma _{1} , \ldots ,\gamma _{{\left| {I_{{jk}} } \right|}} ,\gamma _{1}^{\prime } , \ldots ,\gamma _{{\left| {I_{{jk}} } \right|}}^{\prime } )$$

of length $2 \left| I_{jk}\right|$ called chromosome. Every component of the chromosome, called gene, contains a real number in the unit interval: the first half of the chromosome is used for the sequencing procedure, while the second half is used for the timing procedure.

Let us indicate with $i = 1,\ldots ,\left| I_{jk}\right|$ the surgeries in $I_{jk}$, then $\gamma _i$, $1 \le i \le \left| I_{jk}\right|$, represents the order of patient *i* in the sequence of surgeries, that is the surgeries are sequenced in increasing order of the corresponding gene. By consequence, variables $o_{ii'}$ are fixed according to their definition.

The *i*-th gene of the second half $\gamma _{i}'$ is used to determine the slack time after the *i*-th surgery. Here, the slack time corresponds to the duration of the time interval between the end of the *i*-th surgery and the beginning of the subsequent one. If the considered surgery is the last of the OR block, the slack time is considered with respect to the ordinary end of the OR block. The values of genes $\gamma _{i}'$ are re-scaled according to the total idle time during the encoding phase. Without loss of generality, let us suppose that the patient *i* is the *i*-th in the surgeries sequence, otherwise the patients can be renamed after the decoding of the first half of the chromosome. Firstly, the total scheduled idle time

$$G = L_{{jk}} - \sum\limits_{{i \in I_{{jk}} }} {\mu _{i} } ,$$

is computed as the difference between the OR block duration and the sum of the EOTs of the scheduled surgeries. Then, the scheduled start time $t_i$ of the patient in position *i* in the surgeries sequence is computed as follows

$$\begin{aligned} t_{1} = & 0, \\ t_{{i + 1}} = & t_{i} + \mu _{i} + \frac{{\gamma _{{i^{\prime } }} }}{{\sum\limits_{{i^{\prime } = 1}}^{{\left| {I_{{jk}} } \right|}} {\gamma _{{i^{{\prime \prime }} }} } }}G,\quad i = 1, \ldots ,\left| {I_{{jk}} } \right| - 1, \\ \end{aligned}$$

where $t_i + \mu _i$ is the scheduled completion time of the surgery *i*, while the other term is the decoded slack time left after it.

**Fig. 4:**
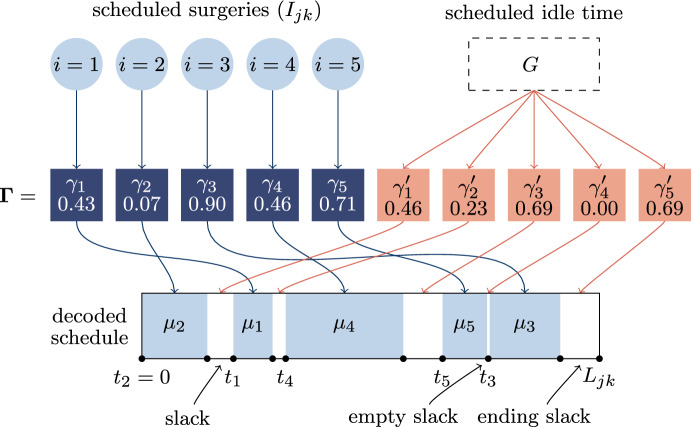
Example of the encoding used in the custom version of the BRKGA

An example of a chromosome with the proposed encoding for an OR block with 5 scheduled patients is represented in Fig. 4. In this particular instance, the first half of genes in the chromosome $\varvec{\Gamma }$ are such that $\gamma _2< \gamma _1< \gamma _4< \gamma _5 < \gamma _3$, then the associated solution of the sequencing procedure is given by the sequence (2, 1, 4, 5, 3). Furthermore, we need to decode the solution of the timing procedure. The surgery $i = 2$ is implicitly scheduled at the OR block start time, that is $t_2 = 0$. To define the scheduled start times of the other 4 surgeries, we consider the second half of genes of $\varvec{\Gamma }$. Since $\gamma _1'$ is equal to 2/9 of the sum $\gamma _1' + \gamma _2' +\gamma _3' +\gamma _4' +\gamma _5'$, then the slack between the first and the second surgery is set equal to 2/9 of the scheduled idle time *G*. Similarly, due to the values of $\gamma _2'$ and $\gamma _3'$ we set a slack of duration *G*/9 between the second and the third surgeries, and a slack of duration *G*/3, respectively. Since it holds that $\gamma _4' = 0$, there is no slack between the fourth and the fifth surgeries, then the scheduled start time of the surgery $i = 3$ is set equal to the scheduled completion time of the surgery $i = 5$. Finally, the last gene $\gamma _5'$ defines the ending slack, that is the scheduled idle time after the last scheduled surgery.

The BRKGA applies evolutionary dynamics over a population of individuals. Each individual of the population is characterized by a chromosome. Each chromosome is associated with a fitness value, defined as follows:

7 $$\text {fitness}(\varvec{t}) = \frac{1}{|\mathcal {S}|}\sum _{\omega \in \mathcal S} Q(\varvec{o}, \varvec{t};\varvec{\xi }(\omega )),$$

which is an approximation of the objective function (2a) through a Monte Carlo simulation over the sample $\mathcal S$. Fitness values are computed through an algorithmic straightforward implementation of $\mathcal B_{jk}^{II}$ that reduces the computational time.

Each gene of each chromosome of each individual of the first population is sampled uniformly at random within the unit interval. For each following generation, that is any following iteration of the algorithm, the BRKGA uses an elitist strategy for generating new offspring. A fixed user-defined fraction of individuals with the lowest fitness values, called elites, is entirely copied to the new population. In this way, good solutions monotonically improve over generations. Then, a user-defined fraction of non-elite individuals, called mutants, is generated uniformly at random and added to the new population. The remaining individuals are generated through a mating mechanism between one member of the elite set and one member of the non-elite set of the previous population. Each gene of the offspring is inherited with a user-defined probability $\tilde{p}$ from the elite parent, and with probability $1 - \tilde{p}$ from the non-elite parent. In this way, the generation of new individuals is biased toward the fittest chromosomes. This process terminates either within a fixed number of generations or a given time limit, returning the individual with the minimum fitness value.

We remark that the proposed chromosome encoding is designed in such a way that it always satisfies constraints (2b)–(2d), guaranteeing the feasibility of the solution associated with every gene. This is a good property that allows us to exploit the whole population to explore the search space and speed up convergence.

## Experimental setup

In this section, we present the creation of the instances that will be used in the computational analysis in Sect. 7. In order to test the proposed stochastic optimization approach on realistic scenarios and to provide reliable managerial insights, we start from data from a real case and we deal with the lack of missing data enhancing it with information available in the literature and data fitting, providing realistic assumptions arising from logical considerations.

### Specialties: MSS, surgical procedures, and distributions

We generated several instances starting from the open data in Mannino et al. (2010) about the major hospital in the city of Oslo (*Sykehuset Asker og Baerum HF*), including information about surgeries of 5 specialties over a period of 3 years: Cardiology (CARDIO), Gastroenterology (GASTRO), Gynecology (GYN), Orthopedics (ORTH), and Urology (URO). We do not include General Medicine (MED) in our experiment due to the low number of surgeries of this specialty, with only the 3% of total cases and an OR block allocated occasionally, which would lead to a trivial scheduling. The patient type (elective or emergency), the surgical specialty, and the start and completion time of the surgery are available in the data set. Therefore, we are able to compute the elective patients’ ROT distribution for each specialty and the daily emergency arrival rate. However, other missing information are usually known in practice when the surgical scheduling is executed, such as the surgical procedure, the EOT, the patient’s age, the OR, and other attributes defining scheduling, cancellation and waiting costs (Azar et al. 2022; Cardoen et al. 2009; Pandit and Carey 2006; Testi et al. 2007; Valente et al. 2009). For this reason, existing data have been integrated through a realistic random generation of the missing ones.

We deduced the MSS, that is assignment of the OR blocks to the 5 specialties over a planning horizon of 5 weekdays from the timestamps of the surgeries in the data set. Since we observed significant variations over the 3-year period, we considered only the last year (2008). For each week and for each day, we compute the total duration of the elective surgeries that happened that day. We assumed the availability of 9 ORs and that the duration of every OR block is 480 min. Thus, we deduced a fixed weekly pattern for the last 8 weeks of 2008. Such a pattern, summarized in Table 3, is the MSS used in our analysis.

**Table 3:** MSS mined from data in Mannino et al. (2010)

OR	Mon	Tue	Wed	Thu	Fri
1	GASTRO	GYN	GYN		
2	GASTRO	GYN	GYN	GYN	GASTRO
3		CARDIO		CARDIO	CARDIO
4		URO		URO	URO
5	ORTH	ORTH		URO	
6		ORTH	ORTH		
7	ORTH		ORTH	ORTH	
8			GASTRO	GASTRO	
9	GYN			GASTRO	

The elective patients’ ROT distribution of every specialty presents several peaks, which suggest the underlying presence of different surgical procedures. In the hypothesis of having the necessary data (which we are artificially reconstructing here), we assume that machine learning techniques can be used to identify a certain number of surgical procedure groups. The surgical procedures of the same groups belong to the same specialty and have similar ROT distribution. Contrarily, surgical procedures in different groups have significantly different ROT distribution, in accordance with the medical literature. For instance, possible urology surgical procedures include dilation of urethra (<30 min), nephrectomy ($\approx$3 h), and total cysectomy (>6 h) (Pandit and Carey 2006). Since the durations of surgical procedure groups can be approximated with a lognormal distibution (Batun et al. 2011; Gul et al. 2011; Strum et al. 2003), we assume that the elective patients’ ROT distribution is a lognormal mixture, where each component is the distribution related to a surgical procedure group. We remark that our purpose is not to predict the surgical procedure durations of the real cases in Mannino et al. (2010), but to generate realistic EOTs consistently with the available ROTs $\rho$. We used the function normalmixEM from the package mixtools (Benaglia et al. 2009) in R 4.1.1 to deduce the distributions that lead to the best fitting Gaussian mixture with respect to the logarithm of the ROTs. We ranged the number *m* of mixtures’ components between 1 and 8, and for each Gaussian component we allowed values of standard deviation in $\{0.15,0.20\}$, which consist in coefficients of variation of the surgical durations equal to about 0.151 and 0.202, allowing us to model two different level of variability. Finally, we selected the mixture with the configuration that approximates better the specialty’s mean and standard deviation.

From the fitted mean and standard deviation of the logarithm of surgery durations, it is possible to deduce the mean $\mu$ (EOT) and the standard deviation $\sigma$ of the surgery duration for each surgical procedure group, while the weights establish their probability to be generated within the specialty. Then, the ROTs of a surgical procedure group have lognormal distribution of parameters $\mu$ and $\sigma$. In Table 4 we list for each specialty $s \in S$ the number $m_s$ of components (surgical procedure groups) of the best-fit mixture, with their frequencies $\varvec{f}$, average values $\varvec{\mu }$, and standard deviations $\varvec{\sigma }$. We remark that such measures are expressed in minutes. In the right column, we illustrate the graph of the duration distribution by representing the logarithm of the ROT on the x-axis. Thus, the probability density function results in a Gaussian mixture. Furthermore, we report the relative errors $\epsilon _{\mu }$ and $\epsilon _{\sigma }$ of mean and standard deviation of the best-fitting mixtures with respect to the empirical distribution of the ROTs.

We obtained 29 different surgical procedure groups among the 5 specialties, with significantly different means and standard deviations. Furthermore, we notice that all the fitting mixtures provide an accurate approximation of the sample mean, that $\epsilon _{\mu }$ is always less than or equal to 0.1%, while the standard deviation varies from an error $\epsilon _{\sigma } = 0.5\%$ for GASTRO up to the $19.8\%$ of CARDIO. However, we deem the mined fitting distributions sufficiently adequate for the purpose of generating realistic scenarios.

**Table 4:** Parameters and density probability function graphs of best fitting mixtures (upper blue curves) and their components (lower green curves) with respect to empirical distributions (histograms). Parameters $\varvec{\mu }$ and $\varvec{\sigma }$ are expressed in minutes

*CARDIO (14.32% of total cases)*	
*m = 7 surgical procedure groups*	
$$\varvec{f} = 0.047, 0.204, 0.172, 0.07, 0.272, 0.083, 0.151$$	
$$\varvec{\mu } = 32.3, 53.4, 67.2, 85.9, 107.9, 136.3, 162.9$$	
$$\varvec{\sigma } = 4.9, 10.8, 13.6, 17.4, 21.8, 20.6, 24.6$$	
$$\epsilon _{\mu } = 0.001$$	
$$\epsilon _{\sigma } = 0.198$$	
*GASTRO (18.76% of total cases)*	
*m = 7 surgical procedure groups*	
$$\varvec{f} = 0.016, 0.089, 0.224, 0.213, 0.243, 0.182, 0.033$$	
$$\varvec{\mu } = 20.0, 41.6, 72.6, 108.7, 159.7, 234.0, 330.4$$	
$$\varvec{\sigma } = 4.0, 8.4, 14.7, 16.4, 24.1, 35.3, 49.8$$	
$$\epsilon _{\mu } = 0.000$$	
$$\epsilon _{\sigma } = 0.005$$	
*GYN (30.43% of total cases)*	
*m = 5 surgical procedure groups*	
$$\varvec{f} = 0.206, 0.15, 0.271, 0.328, 0.045$$	
$$\varvec{\mu } = 24.6, 43.8, 68.2, 124.2, 216.4$$	
$$\varvec{\sigma } = 5.0, 6.6, 10.3, 25.1, 32.6$$	
$$\epsilon _{\mu } = 0.000$$	
$$\epsilon _{\sigma } = 0.010$$	
*ORTH (15.90% of total cases)*	
*m = 5 surgical procedure groups*	
$$\varvec{f} = 0.04, 0.126, 0.239, 0.535, 0.06$$	
$$\varvec{\mu } = 32.9, 63.9, 108.3, 174.3, 243.6$$	
$$\varvec{\sigma } = 6.7, 9.6, 21.9, 26.3, 49.2$$	
$$\epsilon _{\mu } = 0.000$$	
$$\epsilon _{\sigma } = 0.008$$	
*URO (20.59% of total cases)*	
*m = 4 surgical procedure groups*	
$$\varvec{f} = 0.158, 0.451, 0.36, 0.031$$	
$$\varvec{\mu } = 32.1, 58.1, 94.7, 208.8$$	
$$\varvec{\sigma } = 6.5, 11.7, 19.1, 31.5$$	
$$\epsilon _{\mu } = 0.000$$	
$$\epsilon _{\sigma } = 0.041$$	

### Inpatients, outpatients, and emergencies: attributes and costs

While emergencies are indicated within the considered data set with a special attribute, it is not specified if elective surgeries are inpatient or outpatient. In order to have a realistic inpatient and outpatient population with different surgery duration predictability, no-show rates and costs (Wang et al. 2021), the attributes of the elective patients have been generated as listed in Table 5.

**Table 5:** Parameters of inpatient and outpatient population

	Inpatient	Outpatient
	(45% of total cases)	(55% of total cases)
$\mathbb P(\text {type} \, \mid \,\text {LCV})$	0.30	0.70
$\mathbb P(\text {type} \, \mid \, \text {HCV})$	0.70	0.30
No-show rate ($r_i$)	0.08	0.24
Scheduling costs ($c_i^{sched}$)	$\mathcal U$({10,20,...,100})	$\mathcal U$({10,20,...,100})
Cancellation costs ($c_i^{canc}$)	4 $c_i^{sched}$	2 $c_i^{sched}$
Waiting costs ($c_i^{wait}$)	$\mathcal U$([1/180, 1/18])	$\mathcal U$([1/360, 1/36])

Patients with the lower coefficient of variation (LCV = $\sigma _i/\mu _i=0.151$) have been labeled as outpatients with a probability of 0.7 and inpatients with a probability of 0.3. Contrarily, patients with the higher coefficient of variation (HCV = $\sigma _i/\mu _i=0.202$) have been labeled as outpatients with a probability of 0.3 and inpatients with a probability of 0.7. This procedure generated 55% of outpatients and 45% of inpatients, which is an operational context consistent with reality (Omling et al. 2018).

Furthermore, we set the outpatient and inpatient no-show rate to 0.08 and 0.24, which is the average case in the literature, as reported in Wang et al. (2021). The scheduling costs have been generated with uniform distribution over $\{10,20,\ldots ,100\}$ for all patients (i.e., $\Delta = 10$). Then, cancellation costs were set equal to twice the scheduling costs for outpatients and to its quadruple for inpatients. The rationale is that scheduling a patient and canceling them is less preferable than not scheduling them from the beginning. Furthermore, a cancellation of an inpatient could have a higher impact because they could occupy other hospital resources (e.g., upstream units) waiting for rescheduling. By contrast, the direct waiting times of outpatients are less preferable than those of inpatients, as confirmed by the high interest in minimizing waiting times in outpatient clinics. The reason is that a delay may have a major impact on outpatient satisfaction. Therefore, we uniformly sampled a waiting cost (per minute) in [1/180, 1/18] for outpatients and [1/360, 1/36] for inpatients in order to have waiting costs for outpatients that are twice the ones of inpatients on average. The scale of these values has been set in order to have a common sense ratio between the waiting costs and the other two patient costs. For instance, we consider the two following scenarios as equivalent in terms of costs:

*Scenario*1 :  an outpatient *i* with maximum waiting cost and minimum scheduling/cancellation cost is scheduled, but the actual starting time is 3 h after the planned one;
*Scenario*2 :  the same outpatient *i* is not scheduled at all.

However, it is always preferable to delay the surgery of inpatients rather than scheduling and then canceling them, due to the considerations made above.

Two different cases are analyzed to explore two possible cost preferences of the decision maker from an efficiency perspective, that is $c^g = 1/9, c^h = 1/6$, and $c^g = c^h = 1/3$. In the former, the patient-centered costs have a higher impact on the solution, while the facility-centered objectives have greater importance in the latter. The two cases have also a different mutual balancing, which is the same as the two scenarios analyzed in Shehadeh (2022), where the same real case has been considered.

The daily arrival rate of emergency patients is defined as the ratio between the number of emergency patients in the real data set and the number of weekdays. Thus, we computed the probability to insert an emergency surgery within an OR block, that is $p^{em}=0.2$. Setting this parameter, we assumed that emergency surgeries can be performed in all ORs, including those for which an OR block is not planned for the current day (e.g., OR 9 on Monday). Furthermore, the parameters of the lognormal distribution used to generate the emergency surgery durations are set according to the empirical mean and standard deviation of the emergency surgical cases in Mannino et al. (2010), that is $\mu ^{em} = 93$ min and $\sigma ^{em} = 60$ min. Then, the lognormal distribution has been truncated to a maximum of 240 min.

### Instance generation

We generate 10 different instances for each elective surgery waiting list size $\left| W\right| = 500$ and $\left| W\right| = 1100$. Based on average results performed in a preliminary analysis, we need about 3 and 5 weeks to schedule all patients within the fixed MSS, respectively. This means that if the number of executed surgeries per week is approximately the same number of new patients inserted within the waiting list, then the average indirect waiting time will be about 3 and 5 weeks, respectively. The patient population of each instance is randomly generated following the empirical distribution of elective patients among the 5 specialties. At this point, we consider the set $\Pi _s$ of all surgical procedure groups of the patient’s specialty $s \in S$. Then, a procedure group $\pi \in \Pi _s$ is generated with the categorical distribution of parameters $\varvec{f} = (f_1,\ldots ,f_m)$, where $m = \left| \Pi _s\right|$, as reported in Table 4. By consequence, the EOT is set equal to the mean $\mu _{\pi }$ of the lognormal distribution associated with the surgical procedure group $\pi$, which means that in the scenario generation the ROT will have a lognormal distribution of mean $\mu _{\pi }$ and standard deviation $\sigma _{\pi }$.

In all instances, the planning horizon is one week. We set a total duration of $L_{jk} = 480$ min for all OR blocks $(i,j) \in B$, with a maximum overtime of $H = 60$ min per OR block. The same instances have been used to compare different configurations of the model parameters and the proposed methods.

### Tests

For each instance, a sample $\mathcal S$ of size $|\mathcal {S}| = 1000$ has been generated and used for the Monte Carlo sampling, the SAA, the SAA_N_, and the BRKGA. All methods have been implemented in Python 3.10, with Gurobi 10.0.0 for the mathematical programming and the Pymoo Python library (Blank and Deb 2020) for the BRKGA. All tests have been run on an HPC cluster running CentOS, using 4 Intel CPU cores working at 2.1 GHz and 16 GB of RAM to simulate the performance of a standard desktop computer that may be used by an OR planner.

## Computational analysis

We present a computational analysis to evaluate the proposed approaches for the two phases of the stochastic optimization problem introduced in Sect. 3. In Sect. 7.1, we present the results for the advance scheduling model $\mathcal {A}(\alpha ,\varvec{\beta })$ with the Monte Carlo sampling by fixing $\varvec{\beta }$, in order to prove the effectiveness such an approach in for the first phase of our framework and to evaluate the impact of the robustness parameter and the three proxies. The solutions are then used in Sect. 7.2 to compare the performance of the SAA, the SAA_N_, and the BRKGA to solve the allocation scheduling models $\mathcal {B}_{jk}$ in the second phase of the framework. Solutions obtained in these experiments are then combined in Sect. 7.3, where we present the results of the parameter variation illustrated in Fig. 1 discussing the trade-off between robustness and amount of scheduled surgeries, and between the criteria defined through the proxies. Furthermore, in Sect. 7.4 we present an analysis focused on the patient type to provide general managerial insights for the scheduling of inpatients and outpatients within shared ORs. Finally, a sensitivity analysis is presented in Sect. 7.5 to evaluate the level of approximation introduced by the assumption regarding the possibility of inserting at most one emergency patient per OR block.

### Results: advance scheduling

As a first analysis, we solve the model $\mathcal A(\alpha , \varvec{\beta })$ with the Monte Carlo sampling by ranging $\alpha \in \{0.05,0.10,0.15,0.20\}$ to set different levels of robustness and $\varvec{\beta } \in \{(1,0,0),(0,1,0),(0,0,1)\}$ to consider the three proxies one by one and provide a comparison between the different patient mixes determined by them. For each instance we set a total time limit of 1 h, which means that we need half day to compute all the solution for the fixed parameters, that is a reasonable maximum running time for a weekly schedule.

In Table 6, we report results for the two instance sets with a different number of patients ($\left| W\right| =500$ and $\left| W\right| =1100$) to be scheduled by varying the model parameters. Model $\mathcal A(\alpha , \varvec{\beta })$ has been solved by its decomposition in independent subproblems for the 5 specialties ($\mathcal A_s(\alpha , \varvec{\beta })$, with $s \in$ {CARDIO, GASTRO, GYN, ORTH, URO}), then the objective function value (o.f.) has been computed by summing the objective function values of the 5 specialties and the gap (gap) has been computed accordingly. Firstly, we can notice that the use of a general-purpose solver like Gurobi is appropriate, although Monte Carlo sampling increases the computational complexity of the programming model. Average gaps range indeed between 0.26% up to 6.65%, with a general pattern that indicates a greater complexity for lower values of $\alpha$. For the same reason, when $\alpha$ increases the solver is able to find the optimal solution of some instances within the given time limit. Then, the average running time (secs) decreases, accordingly.

**Table 6:** Results solving $\mathcal A(\alpha , \varvec{\beta })$ with different model parameters (average values over 10 instances)

										Sched. patients		
$\vert W \vert$	$\alpha$	$\beta _1$	$\beta _2$	$\beta _3$	o.f.	P1	P2	P3	secs	cost	number	gap
500	0.05	1	0	0	14608.08	8568.00	5.00	185.40	3600	12747	171.8	6.58%
		0	1	0	14607.82	9874.00	0.84	205.60	3600	12743	170.5	6.64%
		0	0	1	14602.16	9402.00	5.00	154.14	3600	12740	171.6	6.65%
	0.10	1	0	0	13820.58	8876.00	4.83	178.34	3335	13535	181.9	1.21%
		0	1	0	13817.58	10410.00	0.90	199.40	3390	13534	182.2	1.23%
		0	0	1	13810.72	10060.20	4.91	99.61	3379	13531	181.9	1.28%
	0.15	1	0	0	13746.33	8748.00	4.91	185.52	3206	13609	182.9	0.65%
		0	1	0	13720.60	10254.00	0.82	208.20	3102	13630	182.0	0.48%
		0	0	1	13725.61	10026.20	5.00	89.37	3152	13616	182.9	0.65%
	0.20	1	0	0	13738.32	8746.00	5.00	172.30	3000	13617	182.5	0.65%
		0	1	0	13719.68	10526.10	0.83	215.50	2875	13631	181.7	0.46%
		0	0	1	13717.53	10126.10	4.92	76.92	3192	13624	183.2	0.59%
1100	0.05	1	0	0	43722.09	11432.00	5.00	196.40	3600	17225	217.5	3.03%
		0	1	0	43722.86	12676.00	1.01	180.50	3600	17218	216.9	3.04%
		0	0	1	43700.26	12424.00	5.00	168.00	3600	17231	217.3	3.03%
	0.10	1	0	0	42637.07	12120.00	5.00	169.00	3144	18311	231.2	0.54%
		0	1	0	42632.28	13624.00	1.05	179.50	3145	18309	231.3	0.54%
		0	0	1	42613.69	13444.00	4.92	105.75	3175	18317	232.2	0.54%
	0.15	1	0	0	42572.77	11920.00	5.00	165.60	2880	18375	231.8	0.38%
		0	1	0	42548.07	13596.10	1.03	187.30	2793	18393	231.4	0.35%
		0	0	1	42528.48	13286.10	5.00	73.13	2865	18402	232.9	0.33%
	0.20	1	0	0	42552.75	11896.00	5.00	164.20	2890	18395	232.0	0.33%
		0	1	0	42518.83	14028.00	1.01	173.10	2799	18422	231.9	0.26%
		0	0	1	42533.54	13394.10	5.00	82.96	2921	18397	232.0	0.33%

**Fig. 5:**
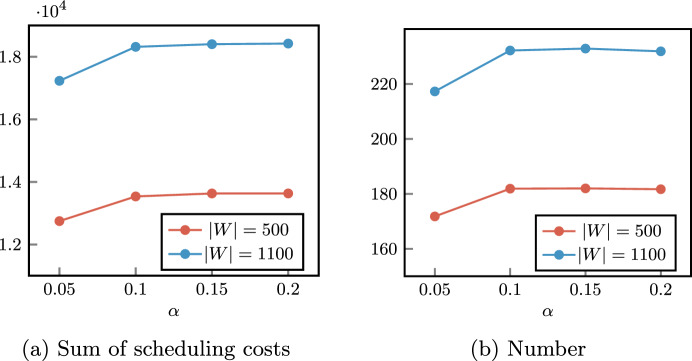
Scheduled patients (sum of the costs of scheduled surgeries and their number) varying the robustness parameter $\alpha$ (best solutions among values of $\varvec{\beta }$)

As expected, the lower the value of $\alpha$, the lower the sum of the scheduling costs of scheduled patients (Sched. patients cost). However, while a significant difference can be observed between $\alpha = 0.05$ and $\alpha = 0.10$, these values tend to settle. This can be better observed in Fig. 5, where for each value of $\alpha$ we considered the best configuration $\varvec{\beta }$ with respect to the total scheduling cost. A counterintuitive result is that, while the cost of scheduled patients slightly increases, the number of scheduled surgeries (Sched. patients number) is almost the same for $\alpha = 0.10,0.15,0.20$, indicating that the chance constraints have a greater effect on the selection of surgeries to be scheduled than on their quantity. Negligible differences can be observed in the objective function values for $\alpha = 0.15$ and $\alpha = 0.20$. This indicates that, around the last value of the robustness parameter, we reach the critical point where the chance constraints (1d) become less strict than the deterministic capacity constraints (1c). Then, for the sack of simplicity, we will show only results for $\alpha \in \{0.05,0.10,0.15\}$ in the second phase of the stochastic optimization approach.

Another expected fact is that different proxies lead to very similar average objective function values, due to the hierarchical modeling of the objectives. Slight fluctuations in the total scheduling costs (column Sched. patients cost) in tests with the same values of *W* and $\alpha$ are mainly due to different complexity caused by different weights in the objective function (1a). However, in the worst case, we have a difference in the total scheduling costs equal to 27, which corresponds to the scheduling of a low-priority surgery.

The effectiveness of the three proxies in determining different types of patient mixes is evident. In columns P1, P2, and P3 we report the average values of the 3 proxies, computed as the sum of the proxy variables $\Gamma ^{canc},\Gamma ^{wait}$, and $\Gamma ^{time}$ over the 5 specialties. Such values clearly show that proxy variables are significantly reduced when the corresponding component in $\varvec{\beta }$ is equal to 1 with respect to 0.

**Fig. 6:**
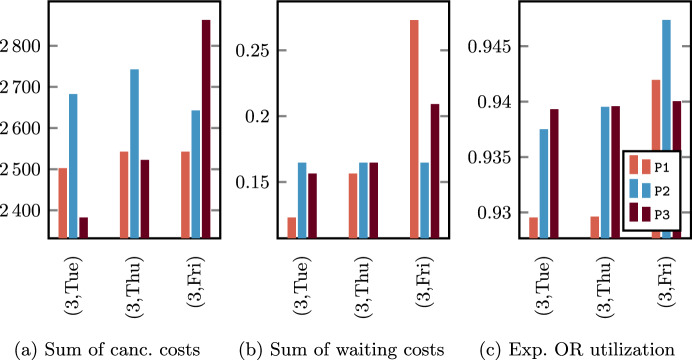
Proxy variables of CARDIO OR blocks for different values of $\varvec{\beta }$ (single instance with $\left| W\right| = 1100$ and $\alpha = 0.1$). P1, P2, and P3 indicate the configurations with $\varvec{\beta }=(1,0,0)$, $\varvec{\beta }=(0,1,0)$, and $\varvec{\beta }=(0,0,1)$, respectively

**Fig. 7:**
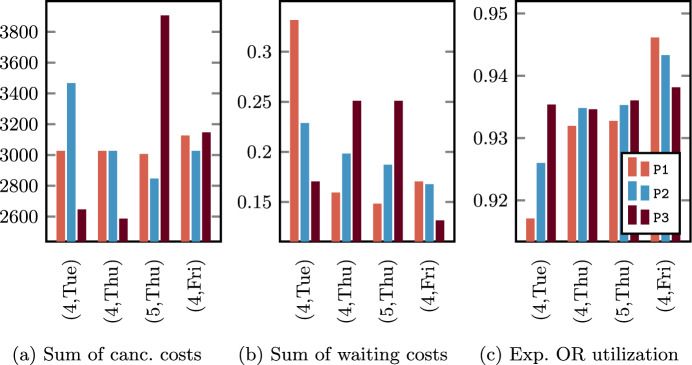
Proxy variables of single URO OR blocks for different values of $\varvec{\beta }$ (single instance with $\left| W\right| = 1100$ and $\alpha = 0.1$). P1, P2, and P3 indicate the configurations with $\varvec{\beta }=(1,0,0)$, $\varvec{\beta }=(0,1,0)$, and $\varvec{\beta }=(0,0,1)$, respectively

The behavior of the three proxies can be further observed in Figs. 6, 7, where we reported the distribution of costs and surgery time among OR blocks for a single instance with $\left| W\right| = 1100$, $\alpha = 0.1$, and two different specialties. In Figs. 6a and 7a we show the sum of cancellation costs of each OR block of the considered specialties, that is defined as the expression in the argument of maximum function in constraint (1e). Analogously, in Figs. 6b and 7b we represent the sum of waiting costs of each OR block that refers to constraint (1f). Finally, in Figs. 6c and 7c we report the expected OR utilization for every OR block, which is equal to the variable $u_{jk}$ in constraint (1i) divided by the OR block capacity $L_{jk} = 480$.

By enabling the first proxy ($\varvec{\beta } = (1,0,0)$) or the second proxy ($\varvec{\beta } = (0,1,0)$) we notice that the maximum sum of cancellation costs and the maximum sum of waiting costs of surgery within the same OR block are reduced with respect to the other configurations. As a side effect, the distribution of the considered costs is balanced among the OR block of the same specialty. For instance, since the OR utilization is independent of the patients’ cancellation/waiting costs, the balancing of the OR utilization provided by the parameter configuration $\varvec{\beta } = (0,0,1)$ could lead to OR blocks whose patient mix involves a high sum of such costs (e.g., see OR block (3,Fri) for CARDIO in Fig. 6a–b). Similarly, setting $\varvec{\beta }$ to consider only the first proxy could lead to a poor solution with respect to the second proxy (e.g., see OR block (3,Fri) for CARDIO in Fig. 6b or OR block (4,Tue) for URO in Fig. 7b), and viceversa (e.g., see OR block (3,Fri) for CARDIO in Fig. 6b or OR block (4,Tue) for URO in Fig. 7b).

Similar considerations can be made for the third proxy in Figs. 6c, 7c. In the parameter configurations with $\beta _3 = 0$, we can observe that the expected OR utilization ranges in an interval of length equal to approximately the 1%$-$1.5% for CARDIO and 1.5%–3% for URO. When the third proxy is enabled ($\varvec{\beta } = (0,0,1)$) the CCIP model is able to find solutions with an almost perfect balancing. Furthermore, an interesting behavior can be observed in Fig. 6c where the solution in correspondence of $\varvec{\beta } = (1,0,0)$ has a lower estimated OR utilization with respect to that with $\varvec{\beta } = (0,1,0)$ OR block by OR block, and with respect to that with $\varvec{\beta } = (0,0,1)$ on average. This is one of the cases where there exist more solutions with the minimum total scheduling costs at the higher level of the hierarchical function, then the weights of the proxy variables have an impact also on the decision about the patients to be scheduled. As a consequence, we expect that the solution provided by $\varvec{\beta } = (1,0,0)$ for this particular instance will lead to a higher total idle time cost but a lower overtime cost compared to $\varvec{\beta } = (0,1,0)$ and $\varvec{\beta } = (0,0,1)$.

Finally, we observe that for the longer waiting list ($\left| W\right| = 1000$) the best solutions consist of a higher number of scheduled patients, whose sum of scheduling costs is also greater. Although in the optimal solutions for the shorter waiting list ($\left| W\right| = 500$) a substantial fraction of surgeries is not scheduled, the increase of patients to be scheduled allows us to find better solutions because (i) there are more patients with a more advantageous ratio between scheduling cost and surgery duration and (ii) there are more possible patient mixes that comply with the capacity constraints (1c)–(1d).

### Results: allocation scheduling

We compare the effectiveness of SAA, SAA_N_, and BRKGA to solve the SMIP models $\mathcal {B}_{jk}$, with $(j,k) \in B$, starting from the advance schedules found in the experiments discussed in Sect. 7.1, and by considering the two different cost preferences $c_g = 1/9, c_h = 1/6$ and $c_g = c_h = 1/3$. We fix a total time limit of 5 min for solving every method, for an overall time limit of 130 min per parameter configuration to solve the allocation scheduling for all the weekly OR blocks of Table 3. We remark that the common practice is to solve the allocation scheduling day-by-day, which in our experimental setup means a required daily maximum running time of 20–30 min per parameter configuration.

A preliminary tuning procedure through a uniform grid search allowed us to set the BRKGA parameters, that is a population size of 50, a fraction of elite and mutant population of 1/4 and 1/5, respectively, and an elite gene inheritance probability ${\tilde{p}} = 1/2$.

**Fig. 8:**
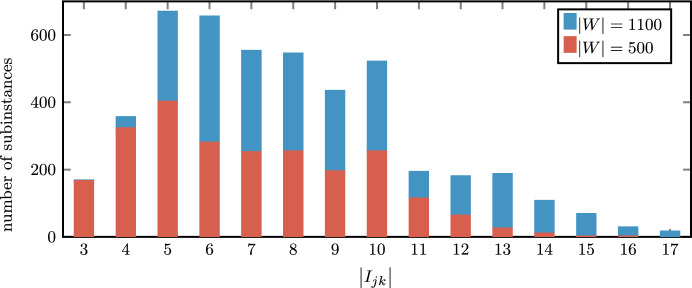
Distribution of the cardinality of the sets $I_{jk}$ (i.e., number of patients scheduled in the same OR block) generated with all parameter configurations

Since the effectiveness of the proposed approaches is strongly dependent on the number $\left| I_{jk}\right|$ of scheduled surgeries in the considered OR block $(j,k) \in B$, we present results divided into groups with similar dimensions of the subinstances used to solve the subproblems $\mathcal B_{jk}$ for all parameter configurations discussed so far (except the case $\alpha = 0.2$ for the reason discussed above). In Fig. 8 we show the distribution of the subinstances with respect to the number of surgeries scheduled in the same OR block, where we can observe a substantial difference between those related to the instance sets with $\left| W\right| = 500$ and $\left| W\right| = 1100$, which leads to the different average number of scheduled patients already discussed in Sect. 7.1. To present meaningful and concise results, all subinstances have been divided into 4 groups with respect to the 4 quartiles determined by the value of $I_{jk}$, that is 3–6, 7–8, 9–10, and 11–17.

Results of the general performance of the three methods are reported in Table 7, with the best value of *N* ($N = 10$) for the SAA_N_. Average values of objective function values and running times are divided into groups of subinstances. The first column (costs) indicates the two cost preferences chosen for this analysis. In the second column ($\left| I_{jk}\right|$) we divide the subinstances with respect to the 4 quartiles determined by the value of $\left| I_{jk}\right|$. Then, for each subset $\mathcal {I}$ of subinstances, the third column (group) considers 3 further subsets of subinstances for a fair comparison: $\mathcal I_{1}$ identifies the subinstances for which all three methods provided feasible solutions, $\mathcal I_{2}$ contains the subinstances for which both the SAA_N_ and the BRKGA found a feasible solution, and $\mathcal I_{3}$ is the set of subinstances such that the BRKGA was able to provide a feasible solution (it holds that $\mathcal I_1 \subset \mathcal I_2 \subseteq \mathcal I_3 \equiv \mathcal I$). The cardinality of groups $\mathcal I_1,\mathcal I_2$, and $\mathcal I_3$ is reported in the fourth column (# inst.).

**Table 7:** Objective function values (o.f.) and running time (secs) of the SAA, the SAA_N_, and the BRKGA for the different cost preferences and groups of subinstances

				SAA		SAA_N_		BRKGA
costs	${\varvec{\left| I_{jk}\right| }}$	group	# inst.	o.f.	secs	o.f.	secs	o.f.
$$\begin{matrix}c_g = 1/9\\ c_h = 1/6\end{matrix}$$	3–6	$\mathcal I_1$	58	28.24	300.00	**22**.**18**	65.08	23.17
		$\mathcal {I}_2 \equiv \mathcal {I}_3$	1848	–	–	**22**.**16**	300.00	22.47
	7–8	$\mathcal I_1$	0	–	–	–	–	–
		$\mathcal {I}_2$	813	–	–	35.56	300.00	**26**.**85**
		$\mathcal {I}_3$	1098	–	–	–	–	**25**.**67**
	9–10	$\mathcal I_1$	0	–	–	–	–	–
		$\mathcal {I}_2$	337	–	–	44.70	300.00	**30**.**79**
		$\mathcal {I}_3$	955	–	–	–	–	**28**.**60**
	11–17	$\mathcal I_1$	0	–	–	–	–	–
		$\mathcal {I}_2$	57	–	–	46.93	300.00	**27**.**63**
		$\mathcal {I}_3$	779	–	–	–	–	**26**.**17**
$$\begin{matrix}c_g = 1/3\\ c_h = 1/3\end{matrix}$$	3–6	$\mathcal I_1$	57	54.04	300.00	**44**.**37**	66.68	45.37
		$\mathcal {I}_2$	1847	–	–	**43**.**97**	300.00	44.12
		$\mathcal {I}_3$	1848	–	–	–	–	**44**.**12**
	7–8	$\mathcal I_1$	0	–	–	–	–	–
		$\mathcal {I}_2$	755	–	–	55.67	300.00	**45**.**02**
		$\mathcal {I}_3$	1098	–	–	–	–	**44**.**19**
	9–10	$\mathcal I_1$	0	–	–	–	–	–
		$\mathcal {I}_2$	350	–	–	63.62	300.00	**47**.**22**
		$\mathcal {I}_3$	955	–	–	–	–	**45**.**94**
	11–17	$\mathcal I_1$	0	–	–	–	–	–
		$\mathcal {I}_2$	63	–	–	61.56	300.00	**43**.**77**
		$\mathcal {I}_3$	779	–	–	–	–	**42**.**93**

For every instance group, the most effective approach is highlighted in bold

**Fig. 9:**
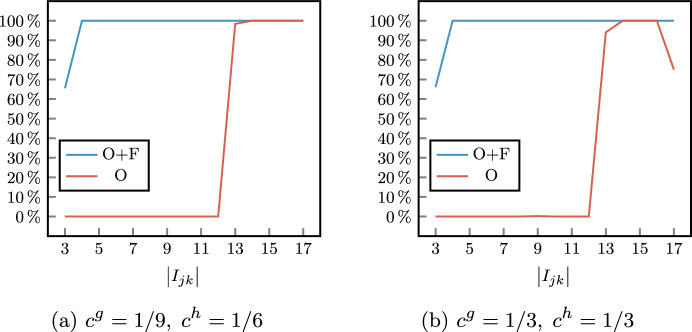
Undesiderable behavior of SAA for the two cost preferences: percentage of instances that caused out-of-memory (O) issues and percentage of instances that caused out-of-memory issues or for which no feasible solution was found within the time limit (O + F)

Contrary to what happens for the advance scheduling model, the SAA is inadequate to solve the proposed SMIP model, with only 1.2% of the subinstances solved, all for lowest values of $\left| I_{jk}\right|$. In all other cases, the ILP model implemented in Gurobi for the SAA method reaches the time limit before finding a feasible solution or the computation ends due to out-of-memory, as shown in Fig. 9 for the two cost preferences and varying the number of scheduled patients. Feasible solutions are found within the time limit only for OR blocks with no more than $\left| I_{jk}\right| = 6$ patients, and the provided solutions are poor with respect to those of the other approaches.

**Fig. 10:**
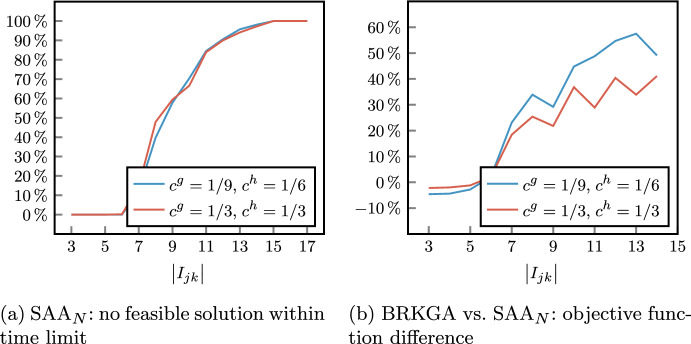
Comparison between SAA_N_ and BRKGA varying the subinstances size $\left| I_{jk}\right|$: ** a** percentage of subinstances for which the SAA_N_ does not find feasible solution within the time limit; ** b** relative difference of the objective function value found with the BRKGA over those with the SAA_N_ (missing values $\left| I_{jk}\right| = 16, 17$ are due to no of feasible solution found within the time limit by the SAA_N_)

The SAA_N_ is instead able to provide solutions for the 64.9% of all subinstances, and for all except one of those with $\left| I_{jk}\right| \le 6$, for which provides results that are slightly better than those of the BRKGA. However, the fraction of solved subproblems decreases when the number of scheduled patients within the OR block increases, as shown in Fig. 10a. Similarly, the quality of the provided feasible solutions gets worse when $\left| I_{jk}\right|$ increases, as shown in Fig. 10b, where we compare the SAA_N_ and the BRKGA in terms of relative difference. This suggests using the proposed SMIP-based approach for OR blocks with a small number of patients, while a metaheuristic is necessary for larger instances.

As highlighted in Sect. 4.3, the encoding of the BRKGA has been designed in such a way to provide only feasible solutions and to exploit the whole running time exploring the search space. On the subinstance groups that allow the comparison with the SAA_N_ (i.e., $\mathcal I_2$), results suggest that the metaheuristic is able to provide near-optimal solutions whose quality is not significantly affected by the instance size. This can be viewed through a comparison with the SAA_N_: while the average objective function values increase when using such a method over increasing value of $\left| I_{jk}\right|$, the BRKGA provides solutions with small fluctuations. This seems to mean that the number of surgeries to be executed within the same OR block does not significantly affect the weighted sum of idle time, overtime, waiting, and cancellation costs. Consequently, the higher the number of patients, the higher the difference between the average objective function values of the SAA_N_ and the BRKGA. No relevant difference emerges between the two different cost preferences.

### Trade-off among objectives: parameter variation

We execute a parameter variation experiment by selecting the solution with the tighter gap of the overall objective function *Z* in (4) for every single instance after solving all the defined subproblems. For every instance and specialty, we get 9 different solutions of the assignment procedure, with different levels of robustness and patient mix within the OR blocks, by using the CCIP model $\mathcal A(\alpha ,\varvec{\beta })$ with three different values of $\alpha$ and three different values of $\varvec{\beta }$, as reported in Table 8. We combine each of those solutions with the best obtained by solving the SMIP model $\mathcal B_{jk}$ through the proposed approaches (i.e., between the SAA_N_ and the BRKGA). At the end, for every OR block we obtain 9 alternative final surgical schedules. Among those, we choose the one with the minimum value of *Z*, that is the minimum weighted sum of the 5 defined costs (scheduling, cancellation, waiting, idle time, and overtime).

**Table 8:** IDs and parameters’ values of the configurations set for the parameter variation

ID	A1	A2	A3	B1	B2	B3	C1	C2	C3
$\alpha$	0.05	0.05	0.05	0.10	0.10	0.10	0.15	0.15	0.15
$\varvec{\beta }$	(1,0,0)	(0,1,0)	(0,0,1)	(1,0,0)	(0,1,0)	(0,0,1)	(1,0,0)	(0,1,0)	(0,0,1)

**Fig. 11:**
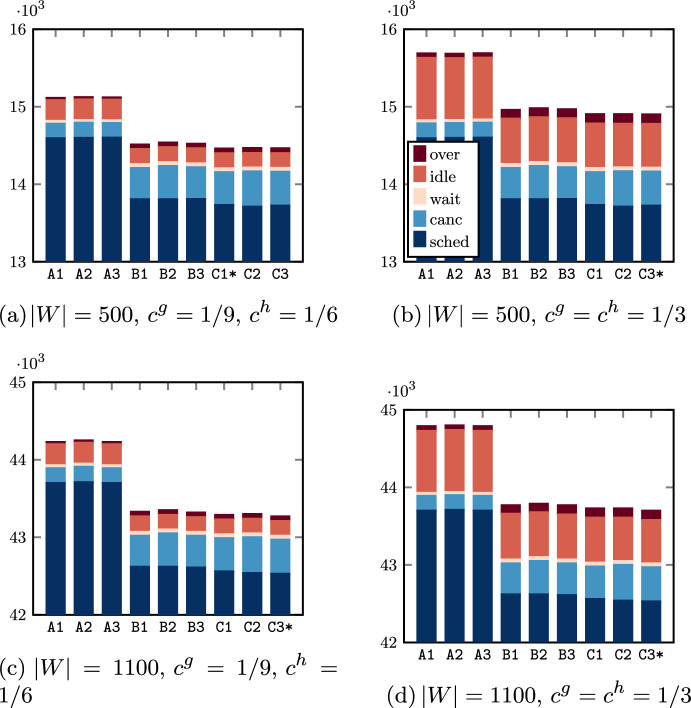
Comparing the 5 different costs and the overall objective function value *Z* for different model parameter configurations (best is marked with symbol *)

In Fig. 11 we show the value of *Z* decomposed into the five different types of costs for the parameter configurations in Table 8, the two instance sets, and the two different cost preferences. The robustness parameter $\alpha$ gives the most important contribution in determining the better parameter configurations: the lower the robustness, the lower the overall objective function value *Z*. In fact, differences in the total scheduling cost between more and less robust advance schedules are not sufficiently compensated by lower overtime and cancellation costs got in the allocation schedules. Furthermore, small differences are obtained by enabling the three different proxies. Fixed the value of $\alpha$, the best configuration is always one between $\varvec{\beta } = (1,0,0)$ and $\varvec{\beta } = (0,0,1)$ due to the reduction of the cancellation costs, which are the ones with the higher variability among configurations. As a consequence, the configuration $\alpha = 0.15$ and $\varvec{\beta } = (1,0,0)$ is the best for the instance set with $\left| W\right| = 500$ and cost preference $c^g=1/9, c^h=1/6$, while $\alpha = 0.15$ and $\varvec{\beta } = (0,0,1)$ provides better results in all other cases.

**Fig. 12:**
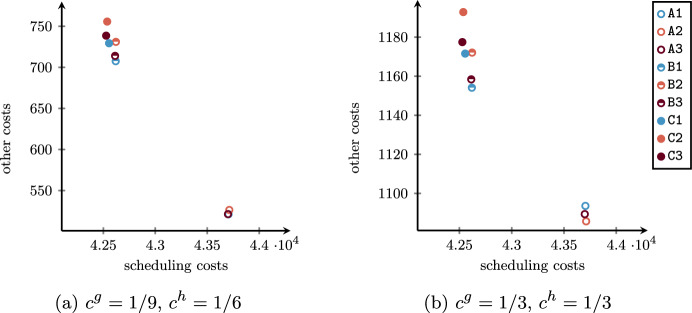
Trade-off between scheduling costs and other costs ($\left| W\right| = 1100$)

**Fig. 13:**
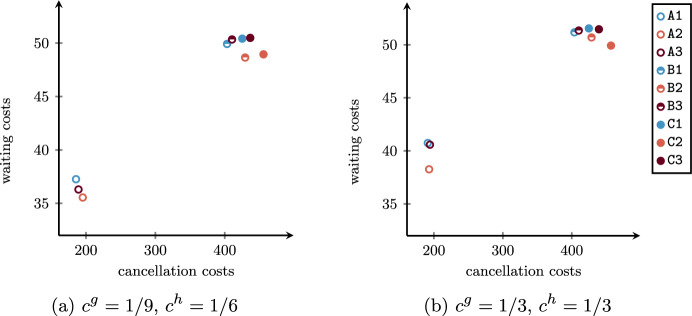
Trade-off between cancellation costs and waiting costs ($\left| W\right| = 1100$)

**Fig. 14:**
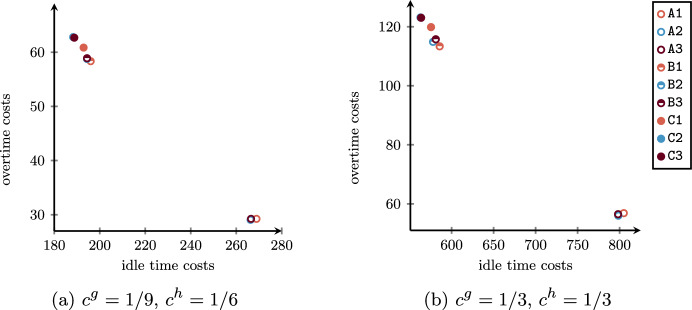
Trade-off between idle time costs and overtime costs

We highlight the differences between final solutions provided by the use of different proxies in the advance allocation model in Figs. 12, 13, and 14, by reporting results of the instance set with $\left| W\right| = 1100$ as representative of the general behavior.

In Fig. 12 we represent the higher level of the hierarchical objective function of $\mathcal A(\alpha ,\varvec{\beta })$ on the x-axis (scheduling costs) and the objective function of $\mathcal B$ on the y-axis (other costs) whose sum is *Z*. Here, the trade-off between the minimization of the indirect waiting time and the ensemble of other objectives is more evident: for a given value of $\varvec{\beta }$, it is not possible to identify a value of $\alpha$ that is Pareto-dominated by another. Conversely, different values of $\alpha$ lead to a different configuration of $\varvec{\beta }$ that minimizes the overall objective function. By enabling the first proxy ($\varvec{\beta } = (1,0,0)$), we minimize the objective function value of $\mathcal B$ for $\alpha \ge 0.15$, but not for $\alpha = 0.05$ (e.g., see Fig. 12(b)).

Nevertheless, it is important to highlight that also for the higher values of $\alpha$, the enabling of the third proxy ($\varvec{\beta } = (0,0,1)$) provides a lower overall objective function value. Theoretically, the hierarchical objective function of model $\mathcal A(\alpha ,\varvec{\beta })$ should always provide the same scheduling costs for a fixed value of $\alpha$. However, the high complexity of the advance scheduling model and the setting of a time limit due to operational reasons lead to different scheduling costs. Consequently, although the advance schedule obtained enabling the first proxy seems to perform better in the allocation scheduling phase, at the end of the day it is more convenient to adopt the third one because it provides lower overall costs. We could define this phenomenon as the *cost of complexity*, which leads to choosing a parameter configuration that is conditioned by the fixed time limit and the complexity given by the different objective function coefficients.

The positive impact of robustness over the direct waiting time and the frequency of cancellations can be observed in Fig. 13. Restricting the evaluation on these two patient-centered indices, each of the parameter configurations with the highest robustness ($\alpha = 0.05$) is Pareto-optimal for the first cost preference (Fig. 13a), with the third proxy that is a compromise between the other two. When $\alpha$ is fixed, the same trade-off among proxies occurs in other cases (e.g., see configurations C1, C2, and C3 in Fig. 13b), while in other cases the solution provided by enabling the third proxy is Pareto-dominated by one of those obtained from the other two proxies (e.g., in both graphs in Fig. 13, B3 is dominated by B1).

Since we assigned a significantly higher weight to cancellations with respect to reasonable direct waiting times and because of a larger order of magnitude of the interval in which the resulting costs range, the first proxy has a more important impact on both the objective function of $\mathcal {B}$ and the overall objective function *Z*. We would say that in general waiting costs are not able to cope with cancellation costs, so when both objectives are optimized simultaneously, an implicit hierarchy is created between them.

With greater reason, the minimization of both direct and indirect waiting times could make sense only in a hierarchical way when inpatients and outpatients share ORs in operational contexts similar to the one considered in our analysis. Since very long direct waiting times are needed to be reasonably compared to the non-scheduling of a patient (i.e., to a higher indirect waiting time), it is unlikely to find two Pareto-optimal solutions with different scheduling costs. We expect that this can be extended to many operational contexts in public health systems unless the unlikely situation in which available OR capacity or overtime is sufficient to guarantee very short waiting lists.

Finally, the two facility-centered indices are represented on the two axis of graphs in Fig. 14. As expected, a higher idle time and lower overtime correspond to a more robust advance schedule. Conversely, when we weaken the chance constraints 1d by increasing $\alpha$, there is a higher expected overtime but the ordinary OR block duration is also exploited more efficiently. A further contribution to this strong trade-off is also given by the parameter vector $\varvec{\beta }$. When $\alpha \ge 0.15$, the first proxy promotes more idle time and less overtime than the other two proxies, which almost completely overlap.

We observe that the third proxy, which balances the expected surgery time among the OR blocks of the same specialty, does not outperform the other two proxies with respect to idle time and overtime. Although the balancing of the cancellation costs and waiting costs has not been designed to act on these two objectives, it emerges that such proxies offer a good balance of the workload among OR blocks as a side effect. Nevertheless, the configuration $\varvec{\beta } = (0,0,1)$ is able to provide the minimum value of *Z* in 3 out of 4 of the instance sets analyzed.

**Fig. 15:**
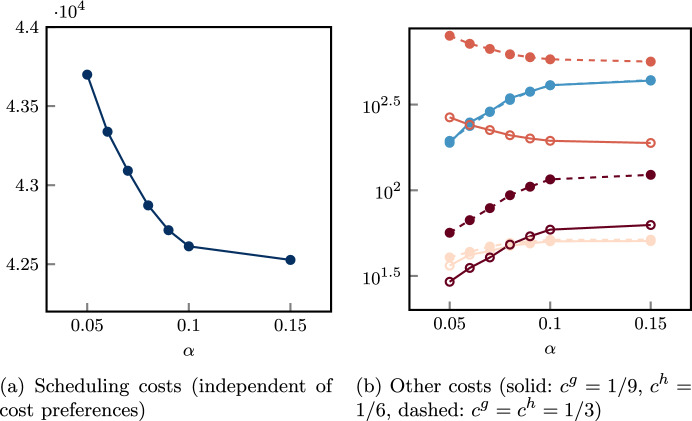
Impact of robustness – further values of $\alpha$ ($\left| W\right| = 1100$, $\varvec{\beta } = (0,0,1)$). Curve colors refer to costs as in the legend of Fig. 11

Finally, we design further parameter configurations by fixing $\varvec{\beta } = (0,0,1)$ (i.e., the best configuration for instance sets with $\left| W\right| = 1100$) and ranging $\alpha \in [0.05,0.1]$ with step 0.01. This choice is guided by the observation of rather similar behaviors in the costs determined with $\alpha = 0.1$ and $\alpha = 0.15$ compared to the substantial difference with $\alpha = 0.05$. Therefore, we enhance our analysis by setting further robustness levels that allow us to evaluate in more detail how the different costs evolve before stabilizing. Results are illustrated in Fig. 15, where we can observe a monotonic trend for all considered criteria in our multi-objective optimization approach.

### Inpatient and outpatients: general insights

Starting from the best parameter configurations found in Sect. 7.3, we can analyze decisions concerning the selection, the mix, and the sequencing of the two different types of elective patients, that is inpatients and outpatients. We report results about the instance sets with $\left| W\right| = 1100$, but similar conclusions can be made for $\left| W\right| = 500$. In Table 9 we report indices that give us summarizing information about the selection and the mix of patients within the OR blocks.

**Table 9:** Schedule indices of the best parameter configuration ($\left| W\right| = 1100$)

Cost preference	$f_{in}$	$f_{out}$	GI_avg_	$\widehat{\text {GI}}$	$q_{in}^{avg}$ (min)	$q_{out}^{avg}$ (min)
$c^g = 1/9, \, c^h = 1/6$	0.5554	0.4446	0.4352	0.8813	118.0	322.7
$c^g = c^h = 1/3$					120.5	322.7

**Fig. 16:**
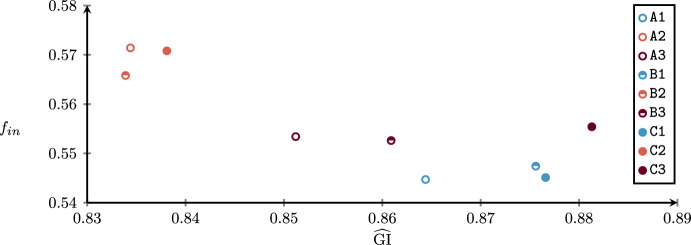
Comparing the fraction of the scheduled inpatients and the normalized average GI of all parameters configurations

The first indices are the fraction of inpatients $f_{in}$ and outpatients $f_{out}$ over the total, which deviates slightly from their distribution within instances to the advantage of the former. Furthermore, we compute the average Gini Index (GI), which is used in machine learning to evaluate the impurity of a set whose elements can have different labels. Given an OR block $(j,k) \in B$, such an index is computed as follow

$$GI_{{jk}} = 1 - \left( {\frac{{n_{{in}} }}{{\left| {I_{{jk}} } \right|}}} \right)^{2} - \left( {\frac{{n_{{out}} }}{{\left| {I_{{jk}} } \right|}}} \right)^{2} ,$$

where $n_{in}$ are $n_{out}$ the number of inpatients and outpatients scheduled in the OR block (*j*, *k*), with $n_{in}+n_{out} = \left| I_{jk}\right|$. Then, we compute the average GI over the whole set *B* and we get the value GI$_{avg} \in [0,1/2]$, which is equal to 0 if the two classes of patients are perfectly separated (i.e., inpatients and outpatients are never scheduled into the same OR block) and rise up to 1/2 when they are mixed. However, since the two classes of patients do not have the same cardinality, we can not have 1/2 as the maximum value of GI, then we computed the value GI$_{max} \le 1/2$ as an upper bound of the maximum average GI, that is in correspondence of $n_{in} = f_{in}\left| I_{jk}\right|$ for each $(j,k)\in B$ (it is an upper bound because the quantity $f_{in}\left| I_{jk}\right|$ can be fractional, while $n_{in}\in \mathbb {N}_0$). Finally, we compute the normalized index $\widehat{\text {GI}} = \text {GI}_{avg} / \text {GI}_{max}$ that represents an impurity index taking into account the distribution of the two scheduled patient classes. As a result, we obtain an average GI equal to 0.4352 and $\widehat{\text {GI}} = 0.88$, which indicate an almost perfect mix of inpatients and outpatients in all the OR blocks. Furthermore, in Fig. 16 we represents the values of $f_{in}$ and $\widehat{\text {GI}}$ corresponding to all parameter configurations over the same instance set. Interestingly, the best configuration (C3) is also the one with the highest value of $\widehat{\text {GI}}$, suggesting that an adequate balancing of inpatients and outpatients in the same OR block provides better performance than allocating different OR blocks to the two class of surgeries. More generally, we can observe that higher levels of robustness lead to lower GIs, while proxies that promote the minimization of cancellation costs and waiting costs lead to a higher and a lower number of scheduled inpatients, respectively. In general, results suggest that a grouping policy that separates inpatient and outpatient surgeries in dedicated OR block is far from being a good strategy with respect to the considered criteria and their weights.

**Fig. 17:**
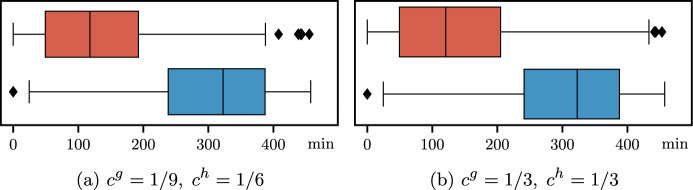
Boxplots representing scheduled starting time for inpatients and outpatients

The last managerial insight emerging from our analysis is about the sequencing and the timing of the two classes of patients. The average scheduled starting times $q_{in}^{avg}$ and $q_{out}^{avg}$ of inpatients and outpatients are very different, suggesting a strong preference for scheduling inpatients at the beginning of the OR block and outpatients in the last positions. This behavior can be observed more in detail in Fig. 17, where the two distributions of the scheduled starting time are significantly different. This can be motivated by the unbalanced weights of cancellations and direct waiting time already discussed and shown in Fig. 13. Since surgeries scheduled at the end of the OR block are affected by both a higher probability of cancellation and a higher expected direct waiting time, the higher costs of cancellations with respect to the waiting costs promote the precedence of inpatients over outpatients. The effect is similar to that of pooling, which is a common strategy used to sequence surgeries with different characteristics (Wang et al. 2022). Furthermore, the pooling effect is slightly attenuated by increasing idle time and overtime costs (e.g., comparing the boxplots of $c^g = c^h = 1/3$ with those of $c^g = 1/9, c^h = 1/6$), which reduces the weight of cancellations with respect to the overall objective function *Z*.

**Fig. 18:**
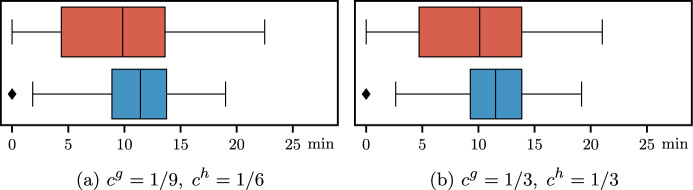
Boxplots representing direct waiting time for inpatients and outpatients

**Fig. 19:**
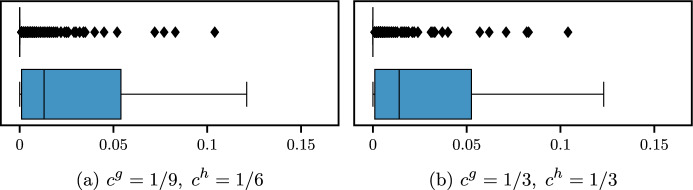
Boxplots representing probability of cancellation for inpatients and outpatients

The impact of positioning inpatients before outpatients in the same OR blocks on waiting times and cancellations is shown in Figs. 18, 19, respectively. Although the average direct waiting times of inpatients and outpatients differ by only 2 min, the distributions are significantly different, with a higher variance in the direct waiting times of inpatients. This counter-intuitive behavior could be caused by the impact of the insertion of emergency patients. Although they have an immediate consequence on patients scheduled immediately after their arrival, the delay caused could be mitigated by slack times and no-shows. As a result, patients at the end of the session (usually outpatients) could benefit from this mitigation, while there is less margin for previous ones.

Contrarily, an expected consequence arises about the probability of surgeries being canceled. As it can be observed in Fig. 19, such a probability is significantly higher for outpatient surgeries compared to inpatient surgeries, which is always negligible except for several outliers.

### Emergency patients: simulation

In this section we perform an analysis to provide: (i) a comparison between the average waiting time of emergency patients when they are inserted in OR block assigned to different specialties and within a schedule with different levels of robustness, and (ii) a measure of the impact of relaxing the assumption about the insertion of at most one emergency patient per OR block. The second analysis has been made through a straightforward DES model in Python 3.10, where emergency patients are generated in accordance with the assumption of the presented model or with a Poisson distribution with the same mean of the Bernoulli distribution described in Sect. 6.1. As well as in the previous operational context, all emergency patients are inserted with the *as soon as possible* policy within the schedule computed under the assumption of having at most one emergency patient.

Results in Tables 10 and 11 for the two cost preferences show how robust solutions lead to slightly shorter waiting times for emergency surgeries. This is due to a higher idle time, during which the emergency patient could arrive and be inserted without waiting for the end of the current surgery in the assigned OR block. Furthermore, such waiting times slightly increase when it is allowed insertion of more than one emergency patient per OR block, with the insertion of the first emergency patient which causes a longer expected wait for the following ones.

Finally, we observe that the assumption about the insertion of at most one emergency surgery per OR block does not have a large impact on the quality of the solutions. The worsening of the objective function value of the allocation scheduling SMIP model $\mathcal B$ (column o.f.) is increased by about 2.5 units, mainly due to over time costs, and it is quite constant among the different parameter configurations, without having a significant impact on the overall performance.

**Table 10:** Performance with at most one emergency patient per OR block (Bernoulli) or more (Poisson): average values of instances with $\left| W\right| = 1100$, $c^{g}=1/9$, and $c^{h}=1/6$

ID	Bernoulli						Poisson					
	o.f.	over	idle	canc	wait	emerg	o.f.	over	idle	canc	wait	emerg
A1	20.97	1.09	10.38	8.07	1.43	26.02	23.44	1.13	10.44	10.49	1.38	28.59
A2	21.57	1.08	10.30	8.84	1.36	25.74	23.81	1.11	10.39	11.03	1.33	28.59
A3	21.30	1.09	10.31	8.51	1.39	25.92	23.62	1.12	10.37	10.77	1.36	28.53
B1	28.41	2.18	7.59	16.74	1.98	29.00	30.67	2.21	7.62	18.97	1.87	31.61
B2	29.21	2.19	7.50	17.68	1.85	28.55	31.54	2.21	7.53	19.99	1.81	31.42
B3	28.58	2.20	7.51	16.96	1.91	29.01	31.01	2.24	7.55	19.36	1.87	31.59
C1	29.25	2.29	7.43	17.61	1.92	29.00	31.46	2.31	7.47	19.81	1.87	31.52
C2	30.83	2.36	7.29	19.31	1.87	29.57	33.10	2.39	7.32	21.58	1.82	32.30
C3	29.60	2.34	7.30	18.03	1.93	29.40	32.11	2.39	7.33	20.51	1.89	31.60

Columns “emerg” indicate average waiting time (min) of emergency patients

**Table 11:** Performance with at most one emergency patient per OR block (Bernoulli) or more (Poisson): average values of instances with $\left| W\right| = 1100$, $c^{g}=1/3$, and $c^{h}=1/3$

ID	Bernoulli						Poisson					
	o.f.	over	idle	canc	wait	emerg	o.f.	over	idle	canc	wait	emerg
A1	42.61	2.12	31.05	7.89	1.55	26.10	45.15	2.22	31.22	10.21	1.50	28.64
A2	42.91	2.11	30.80	8.53	1.47	25.65	45.47	2.17	30.97	10.90	1.43	28.58
A3	42.82	2.11	30.84	8.33	1.54	25.83	45.33	2.19	31.02	10.61	1.50	28.41
B1	45.65	4.30	22.69	16.70	1.96	29.08	48.05	4.36	22.80	18.97	1.92	31.50
B2	46.23	4.33	22.42	17.55	1.93	28.67	48.69	4.38	22.53	19.89	1.89	31.48
B3	45.67	4.35	22.46	16.89	1.97	28.79	48.31	4.43	22.57	19.39	1.93	31.54
C1	46.26	4.54	22.23	17.51	1.97	28.93	48.54	4.58	22.34	19.70	1.93	31.39
C2	47.59	4.65	21.75	19.27	1.92	29.54	49.93	4.70	21.86	21.50	1.87	32.12
C3	46.38	4.65	21.84	17.92	1.97	29.26	49.09	4.73	21.93	20.50	1.93	31.68

Columns “emerg” indicate average waiting time (min) of emergency patients

## Conclusions

In this paper we presented a two-step stochastic optimization framework for the surgical schedule problem of operating theaters from the perspective of elective patients, modeling and analyzing two particular classes of patients with different characteristics and preferences, that is inpatients and outpatients. Three different uncertainty factors were considered, namely the duration of the surgeries, the arrival of emergency patients, and no-shows. The aim of the new proposed approach is to provide a decision support tool for the operational context in which these two classes of patients shares ORs and to present a quantitative analysis in which research questions emerged in recent literature reviews of OR planning and scheduling.

A CCIP model has been proposed to solve allocation scheduling by setting parameters that determine the robustness of the schedule with respect to cancellations and the OR block patient mix. Starting from a solution obtained in the first phase, a SMIP model allows us to fix the sequencing and the timing of the surgical procedures. At the end, among all the parameter configurations, we choose the one that minimizes an objective function including costs associated with direct and indirect waiting time, cancellations, idle time, and overtime.

Monte Carlo sampling has been adopted to solve the two proposed mathematical programming models with a general-purpose solver. While it resulted to be a methodology capable of providing near-optimal advance scheduling solutions in reasonable times with respect to the application context, the classic SAA proved to be completely inadequate in solving the allocation scheduling SMIP model, due to its high computational complexity. Therefore, two alternative techniques have been proposed: the *N*-fold SAA as a variant of SAA that is effective on small-size instances and the BRKGA with a custom encoding that outperforms the other approaches on medium- and large-size instances.

Several interesting results emerged from the quantitative analysis, based on real data from a Norwegian hospital, enriched with a realistic generation of missing but useful information for the purposes of our study. Although the construction of robust surgical schedules effectively guarantees a reduction in cancellation costs, this entails a worsening of the costs associated with indirect waiting time and idle time. This worsening is such as providing solutions that are inefficient and not preferable to those obtained by setting a low robustness level. Consequently, a real trade-off between direct and indirect waiting time seems to be almost absent, since the scheduling costs have an order of magnitude much higher than the waiting costs in common operational contexts. This suggests that when inpatients and outpatients share ORs, indirect and direct waiting times could be optimized in a hierarchical way, with the former at the upper level and the latter at the bottom level.

The analysis of the three different proxies proposed to promote the composition of different patient mixes within the OR blocks allowed us to evaluate the impact of balancing the patients with the highest cancellation and waiting costs between the OR blocks, or to balance the their total expected duration. Once again, direct waiting time turned out to be the least driving criterion, since the best configuration for different instance sets always resulted in the enabling of proxies designed to minimize cancellations and balance idle time and overtime. However, in this case, the orders of magnitude were comparable, and therefore different operational contexts or preferences of the decision-maker could lead to other types of choices. Nonetheless, a general managerial insight concerns the composition of the schedules that have performed better, in which inpatients and outpatients are indifferently scheduled in the same OR blocks, with those of the former type sequenced at the beginning and those of the latter type scheduled at the end, with an effect very similar to a pooling strategy.

Future research developments can follow three different directions. First, the analysis for which the proposed approach was designed can be adapted to real case studies with sufficient information to identify real surgical procedures and their duration. Such information could be exploited by machine learning models for the prediction of the duration of the interventions and of the probability of no-shows of particular classes of patients. New proxies can be identified or the proposed ones can be combined (i.e., through different weights) to define different patient mixes within the OR blocks. Furthermore, the impact of optimizing the OR scheduling week-by-week over time (Addis et al. 2016; Aringhieri et al. 2018) with the proposed stochastic optimization framework deserves to be studied.

From a modeling point of view, a further generalization of the stochastic optimization framework could include the availability of downstream and upstream resources, whose allocation to inpatients and outpatients follows different needs. Furthermore, different overtime allocation policies could be implemented, as well as the impact of other non-elective patient admission rules (e.g., see Duma and Aringhieri (2018, 2019); Gökalp et al. (2023); Spratt and Kozan (2021)) could be investigated. From a methodological perspective, the proposed approach can be extended by simultaneously solving advance scheduling and allocation scheduling by designing metaheuristics able to address the high complexity of the problem.

## Appendix A: Linear formulation of the SMIP model $\mathcal {B}_{ij}^{II}(\omega )$

In this section we report linearization formulation of non-linear constraints in the SMIP model $\mathcal {B}_{ij}^{II}(\omega )$. Big-M and a small constant $\epsilon > 0$ are used in several constraints, while auxiliary variables are defined for each specific group. Linearization of constraints (3p) is trivial, then we omit it. Constraints (3b) are linearized by introducing the following constraints, in which auxiliary variables $\gamma _i,\hat{\gamma }_i \in \{0, 1\}$ are used:

$$\begin{aligned}&c_i \ge t_{i'} - M(1 - \gamma _i) - M(1 - o_{ii'}),&\;\; i, i', i \ne i^\prime \in I_{jk}, \\&c_i \le t_{i'} + M\gamma _i - \epsilon (1 - \gamma _i) + M (1 - o_{ii'}),&\;\; i, i', i \ne i^\prime \in I_{jk}, \\&q_{i'}\ge t_{i'} - M\gamma _i - M (1 - o_{ii'}),&\;\; i, i', i \ne i^\prime \in I_{jk}, \\&q_{i'}\le t_{i'} + M\gamma _i + M (1 - o_{ii'}),&\;\; i, i', i \ne i^\prime \in I_{jk}, \\&q_{i'}\ge c_i - M(1 - \gamma _i) - M (1 - o_{ii'}),&\;\; i, i', i \ne i^\prime \in I_{jk}, \\&q_{i'}\le c_i + M(1 - \gamma _i) + M (1 - o_{ii'}),&\;\; i, i', i \ne i^\prime \in I_{jk}, \\&\hat{c}_i \ge t_{i'} - M(1 - \hat{\gamma }_i) - M(1 - o_{ii'}),&\;\; i, i', i \ne i^\prime \in I_{jk}, \\&\hat{c}_i\le t_{i'} + M\hat{\gamma }_i - \epsilon (1 - \hat{\gamma }_i) + M (1 - o_{ii'}),&\;\; i, i', i \ne i^\prime \in I_{jk}, \\&\hat{q}_{i'}\ge t_{i'} - M\hat{\gamma }_i - M (1 - o_{ii'}),&\;\; i, i', i \ne i^\prime \in I_{jk}, \\&\hat{q}_{i'} \le t_{i'} + M\hat{\gamma }_i + M (1 - o_{ii'}),&\;\; i, i', i \ne i^\prime \in I_{jk}, \\&\hat{q}_{i'}\ge \hat{c}_i - M(1 - \hat{\gamma }_i) - M (1 - o_{ii'}),&\;\; i, i', i \ne i^\prime \in I_{jk}, \\&\hat{q}_{i'} \le \hat{c}_i + M(1 - \hat{\gamma }_i) + M (1 - o_{ii'}),&\;\; i, i', i \ne i^\prime \in I_{jk}. \end{aligned}$$

Constraints (3j) are linearized by introducing the following constraints, in which the auxiliary variables $\Lambda _i^{(1)},\Lambda _i^{(2)} \in \{0,1\}$ are used:

$$\begin{aligned}&\tau _{jk}(\omega ) - \hat{q}_{i'} \ge \epsilon - M \Lambda ^{(2)}_i + \epsilon (1 - \Lambda ^{(2)}_i) - M(1 - o_{ii'}),&\;\; i, i', i \ne i^\prime \in I_{jk}, \\&\tau _{jk}(\omega ) - \hat{q}_{i'} \le \epsilon + M(1- \Lambda ^{(2)}_i) + M(1 - o_{ii'}),&\;\; i, i', i \ne i^\prime \in I_{jk}, \\&\tau _{jk}(\omega ) - \hat{q}_{i'} \ge - M (1 - \Lambda ^{(1)}_i),&\;\; i \in I_{jk}, \\&\tau _{jk}(\omega ) - \hat{q}_{i'} \le M\Lambda ^{(1)}_i - \epsilon (1 - \Lambda ^{(1)}_i),&\;\; i \in I_{jk}, \\&\Lambda ^{(1)}_i + \Lambda ^{(2)}_i \le 1 + e_i,&\;\; i \in I_{jk}, \\&e_i \le \Lambda ^{(\ell )}_i,&\;\; i \in I_{jk}, \ell = 1, 2. \end{aligned}$$

Constraints (3k) are linearized by introducing the following constraints, in which auxiliary variables $\lambda _i^{(e)},\lambda _i^{(\tau )} \in \{0,1\}$ are used:

$$\begin{aligned}&\tau _{jk}(\omega ) \le q_i + \rho _i(\omega ) \theta _i(\omega ) y_i + M \lambda _i^{(\tau )} - \epsilon (1 - \lambda _i^{(\tau )}),&\;\; i \in I_{jk},\\&\tau _{jk}(\omega ) \ge q_i + \rho _i(\omega ) \theta _i(\omega ) y_i - M(1 - \lambda _i^{(\tau )}),&\;\; i \in I_{jk},\\&z_i \le \tau _{jk}(\omega ) - (q_i + \rho _i(\omega ) \theta _i(\omega ) y_i) + M(1 - \lambda _i^{(e)}),&\;\; i \in I_{jk}, \\&z_i \ge \tau _{jk}(\omega ) - (q_i + \rho _i(\omega ) \theta _i(\omega ) y_i) - M(1 - \lambda _i^{(e)}),&\;\; i \in I_{jk}, \\&e_i + \lambda _i^{(\tau )} \le 1 + \lambda _i^{(e)},&\;\; i \in I_{jk}, \\&z_i \le M \lambda _i^{(\tau )},&\;\; i \in I_{jk}. \end{aligned}$$

Finally, constraints (3l) are linearized by introducing the following constraints:

$$\begin{aligned}&\theta _i(\omega ) (q_i + \mu _i ) \le L_{jk} + H + M(1 - y_i),&\;\; i \in I_{jk}, \\&\theta _i(\omega ) (q_i + \mu _i ) \ge L_{jk} + H - My_i + \epsilon (1 - y_i),&\;\; i \in I_{jk}. \end{aligned}$$
